# Synthesis, characterization, and polyester dyeing performance of azo barbituric and thiobarbituric acid disperse dyes

**DOI:** 10.1038/s41598-025-96473-x

**Published:** 2025-04-18

**Authors:** Mohamed A. El-Rahman, Alaa Z. Omar, Alshimaa R. Kandeel, Ezzat A. Hamed, Mohamed A. El-Atawy, Reda M. Keshk

**Affiliations:** 1 Chemistry Department, Faculty of Science, Alexandria University, P.O. 426, Ibrahemia, Alexandria, 21321 Egypt; 2 Chemistry Department, Faculty of Science, Damanhour University, Damanhour, Egypt

**Keywords:** Disperse dye, Azo dye, Barbituric acid, Polyester, Color measurements, Chemistry, Organic chemistry, Chemical synthesis, Theoretical chemistry

## Abstract

This study explores the synthesis, characterization, and application of 23 azo disperse dyes synthesized based on barbituric and thiobarbituric acid derivatives for polyester coloration. The dyes were synthesized via the coupling of diazotized aniline derivatives with barbituric and thiobarbituric acid derivatives. The structures of these synthesized dyes are assigned using FT-IR and^1^H NMR spectroscopy. UV-Vis spectroscopy revealed absorption maxima ranging from 360 to 440 nm, with good correlation between experimental and theoretical values calculated using TD-DFT. The dyes exhibited significant pH-dependent spectral shifts, demonstrating potential as pH indicators. Application to polyester fabrics showed excellent wash fastness (rating 5) for all dyes, with varying results for perspiration, scorch, and light fastness. The dyes demonstrate dye exhaustion ranging from 43.6 to 92.5%, with color strength (K/S) values up to 22.00, while computational insights from DFT calculations provide deep understanding of structure-property relationships. The study demonstrated that substituent type and position significantly influenced dye properties and performance. Notably, dye **17** (4-OCH_3_) exhibited the highest K/S (21.40) and K/S_sum_ (349.05) values, correlating with its low HOMO-LUMO gap (0.1177 eV). These findings contribute to understanding the structure-property relationshipsin azo disperse dyes and their application in polyester dyeing.

**Supplementary Information:**

The online version contains supplementary material available at 10.1038/s41598-025-96473-x.

## Introduction

Disperse dyes are a class of synthetic organic colorants widely used in the textile industry due to their excellent textile properties and high chemical stability. Polyester fabrics have a hydrophobic nature and a highly compact structure and they are dyed using disperse dyes at high temperatures (usually in the range of 115–135 ºC) and high pressure^1,2^. Disperse dyes are essentially non–ionic dyes, exhibiting poor solubility in water and therefore they are applied in the form of water dispersion^3–5^. The development of new disperse dyes with improved properties continues to be a focus of research, driven by the need for more efficient, environmentally friendly, and versatile coloration options for synthetic textiles^6^.

Dyeing of polyester fabric in the water dye bath by the exhaustion process is carried out in a slightly acidic medium. Many disperse dyes degrade if the pH is uncontrolled during aqueous dyeing; some disperse dyes have hydrolysable groups in their molecules, which makes them particularly sensitive to hydrolysis, especially in alkaline medium. The hydrolyzed form of the dye could be of different shade and, in some cases, have a different affinity for polyester fibres than the unhydrolyzed dye. Thus, to minimize the possibility of dye hydrolysis, the dyeing is carried out in a slightly acidic medium, usually in the pH range of 4.5–5.5. According to literature data, acetic acid is generally used for adjusting the dyebath pH value, although a buffer system containing formic acid and ammonium sulfate is used as well^7^.

Azo dyes, characterized by the presence of one or more azo (-N = N-) groups, represent a significant portion of disperse dyes used in the textile industry^8,9^. These dyes are valued for their wide range of colors, good fastness properties, and relatively simple synthesis^10–12^. The azo group’s ability to form Van der Waals forces with polyester fibres contributes to the dyes’ excellent wash fastness and overall performance. The electronic properties of azo dyes, influenced by various substituents, play a crucial role in determining their color, reactivity, and dyeing behavior^13^.

Several studies have explored the synthesis and application of azo disperse dyes for polyester coloration. Traditional disperse dyes, including those based on various heterocyclic moieties have been widely used due to their strong affinity for synthetic fibers and good fastness properties^14,15^. In particular, barbituric and thiobarbituric acid-based dyes have attracted interest due to their ability to form stable tautomeric structures, which influence their spectral and dyeing behaviors^5,16–18^. However, many of these studies have focused on different substituents and their impact on spectral properties, with limited insight into the combined effects of substituent type, dyeing behavior, and electronic properties. This study extends previous research by systematically investigating the synthesis and application of azo disperse dyes incorporating barbituric and thiobarbituric acid derivatives.

Azo barbituric acid and its thio analog derivatives have emerged as promising candidates for disperse dyes due to their unique molecular structure and electronic properties. The barbituric acid moiety, with its multiple carbonyl groups, can participate in various interactions with textile fibers, potentially enhancing dye adsorption, fixation and color fastness. Additionally, the azo group conjugated with the barbituric moiety can exist in different tautomeric forms, contributing to its interesting spectral properties and pH-responsive behavior. Our recent studies^16^ have demonstrated the exceptional color strength and fastness properties of barbituric acid-based chromophores when applied to synthetic fibers. The choice of barbituric and thiobarbituric acid derivatives is particularly advantageous due to their excellent electron-accepting capabilities. Additionally, the azo group conjugated with the barbituric moiety can exist in different tautomeric forms, contributing to its interesting spectral properties and pH-responsive behavior, which has been utilized in developing smart textile materials with sensing capabilities^19^.

Environmental considerations are increasingly important in the development of textile dyes, particularly for synthetic fibers like polyester. Traditional disperse dyes often pose environmental challenges due to their persistence, bioaccumulation potential, and toxicity to aquatic organisms^20^. In contrast, azo disperse dyes based on barbituric and thiobarbituric acid derivatives potentially offer improved environmental profiles^21^. While maintaining excellent coloration properties, these dyes are designed with structural features that may enhance biodegradability through susceptibility to enzymatic cleavage at the azo linkage^22^. Additionally, the incorporation of barbituric and thiobarbituric acid moieties, which are derivatives of naturally occurring pyrimidines, potentially reduces the bioaccumulation and ecotoxicological impact compared to conventional disperse dyes containing more persistent aromatic structures^23^.

The coloration of polyester fabrics with azo disperse dyes is a critical area of study due to its industrial significance and the need for more efficient and environmentally friendly dyeing processes. This study aims to investigate the synthesis and characterization of azo disperse dyes based on barbituric and thiobarbituric acid derivatives to enhance polyester coloration. By strategically incorporating barbituric and thiobarbituric acid derivatives, we demonstrate a rational approach to developing dyes with tunable electronic properties, superior fastness characteristics, and inherent pH-responsive behavior.

Our comprehensive approach encompasses the synthesis and spectroscopic characterization of dyes produced by coupling diazonium salts of substituted anilines with barbituric and its analogue, along with an investigation of their spectral properties, including UV-Vis absorption and pH-dependent behavior. Additionally, we assess their dyeing performance on polyester fabrics, evaluating fastness properties, dye exhaustion, and color strength, and conduct colorimetric analysis using the CIE Lab color space. The exploration of electronic properties through DFT calculations of the titled dyes is conducted by correlating molecular structure with spectral characteristics, dyeing performance, and electronic properties, we seek to establish structure-property relationships that will guide the future design of more efficient disperse dyes for polyester coloration, potentially expanding their applications beyond textile dyeing.

## Results and discussion

### Chemistry

#### Preparation of (*E*)-5-(aryldiazenyl)pyrimidine-2,4,6(1*H*,3*H*,5*H*)-trione 1–18

**Scheme 1:**
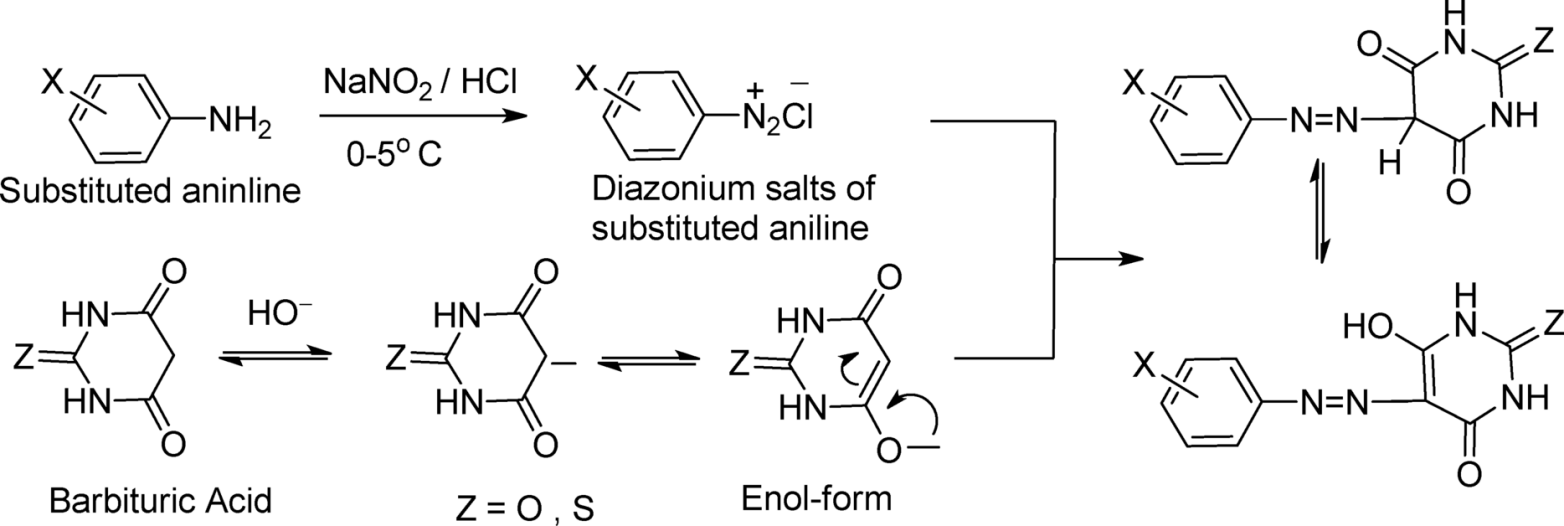
First mechanism for synthesis of disperse azo dyes **1–18**.

**Scheme 2:**
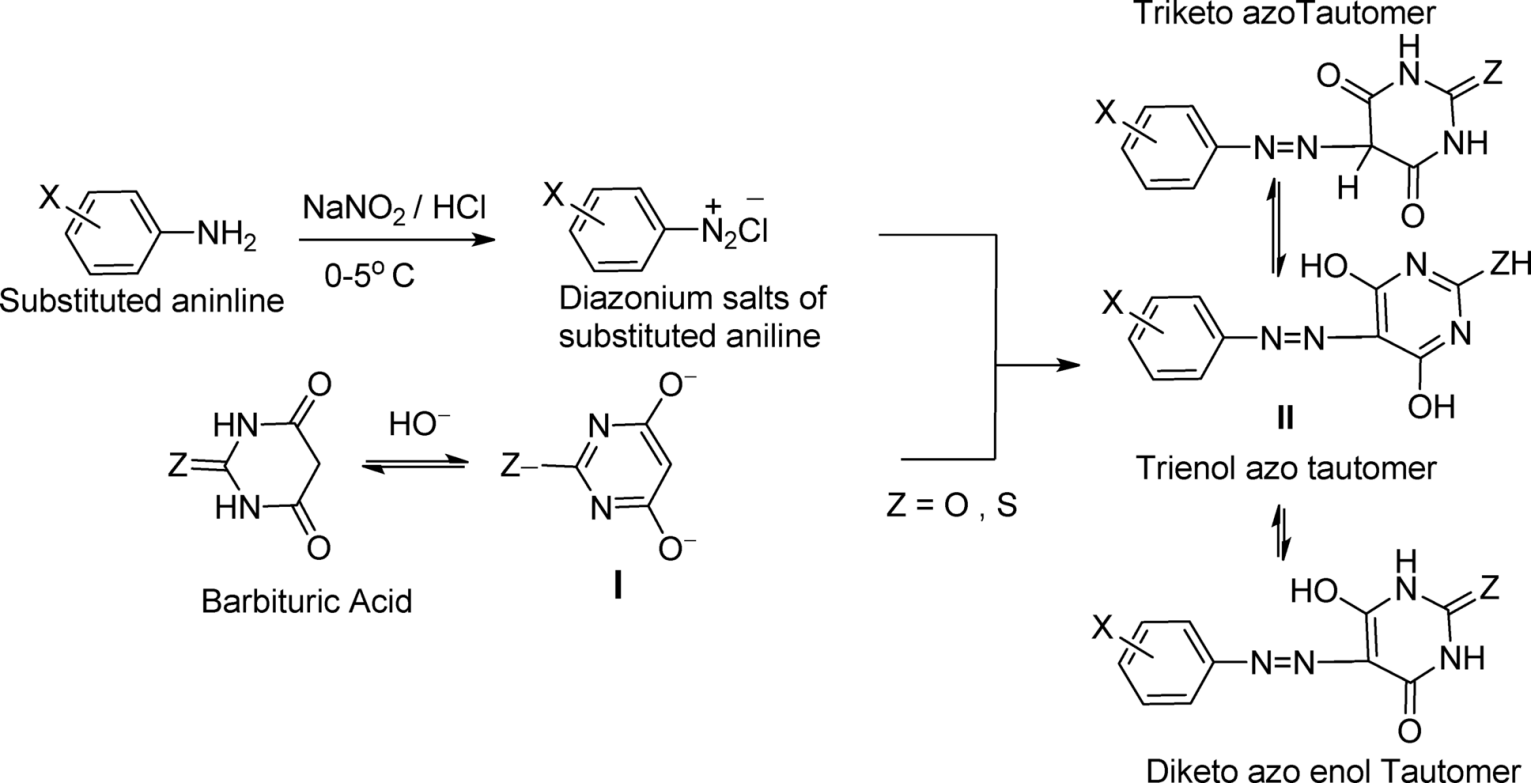
Second mechanism for Synthesis of disperse azo dyes **1–18**.

**Scheme 3:**
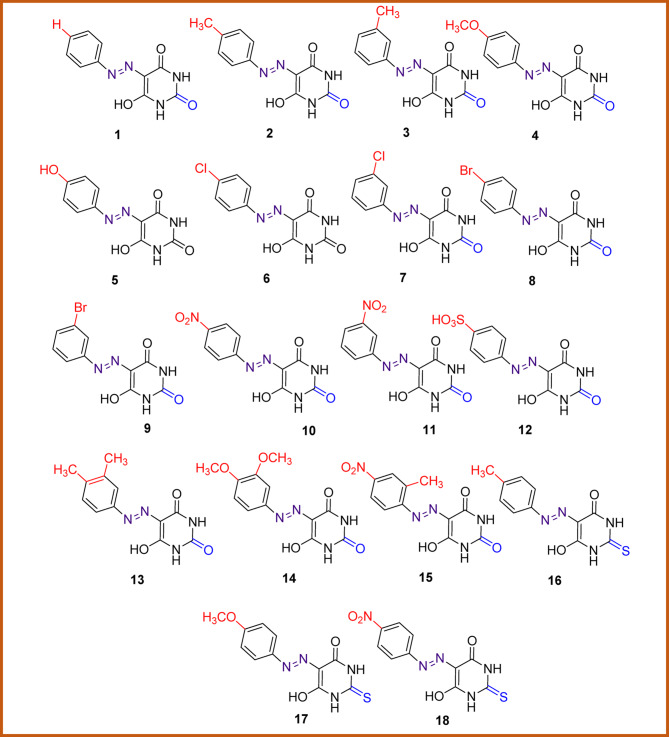
Synthesis of disperse azo dyes **1–18**.

In this work, the reaction occurs in two steps: in the first step, the reaction of substituted anilines (4-CH_3_, 3-CH_3_, 4-OCH_3_, 4-OH, 4-Cl, 3-Cl, 4-Br, 3-Br, 4-NO_2_, 3-NO_2_, 4-SO_3_H, 3,4-di CH_3_, 3,4-di OCH_3_, 2-CH_3_-4-NO_2_) with sodium nitrite occurs in an acidic medium, producing the diazonium salt. In the second step, the diazonium salt is coupled with the barbituric acid and thiobarbituric acid in an alkaline medium to produce azo compound **1–18**, Schemes 1–3.

The spectral data as well as the elemental analysis reveal that the dye was formed by the reaction of barbituric acid with diazotized substituted aniline reagents in a 1:1 ratio, indicating a mono azo dye likely formed through one of the following mechanisms: the first mechanism, barbituric acid (or its thiobarbituric acid counterpart, where Z = O for barbituric acid or Z = S for thiobarbituric acid) first undergoes enolization in the presence of a base, forming a reactive enolate ion. This enolate then reacts with the diazonium salt, produced by diazotizing substituted anilines with sodium nitrite and hydrochloric acid, leading to the formation of azo dyes **1–18**, Scheme 1.

The Second mechanism, barbituric acid or thiobarbituric acid strongly deprotonated at all three positions to generate a pyrimidine-based trianion. This trianion can serve as a powerful nucleophile, allowing it to react effectively with the diazonium salt to create the azo coupling products that can exist in different tautomeric forms: triketo, trienol, or diketo-enol tautomers, depending on the protonation and bonding arrangement. Scheme 2.

**Fig. 1:**
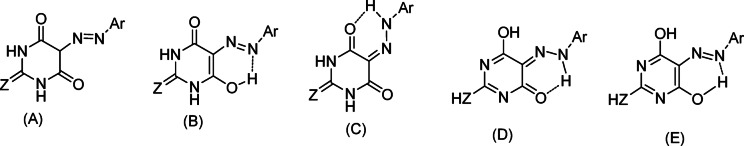
Different tautomeric forms of azo barbituric acid and thiobarbaturic acid.

It is well known that azo barbituric acid and thiobarbituric acid can exist in five tautomeric forms (A to E), as shown in Figure. Namely, triketo-azo tautomer A, diketo-enol azo tautomers B and C, keto-dienol hydrazo tautomer D, and trienol-azo tautomer Eas shown in Fig. 1.

The FT-IR spectra of disperse azo dyes **1–18** showed intense absorption peaks assigned to carbonyl (C = O) groups at ν 1758 − 1701 and 1709 –1654 cm^− 1^. Moreover, FT-IR spectra of dyes **1–18** exhibited small and medium absorption peaks at ν 3530 − 3121 and 1592 –1512 cm^− 1^, corresponding to stretching vibrations of imino (N-H) and azo (N = N) groups^24^, respectively. This resultsuggests that the tautomeric equilibrium of these dyes is in favour of the triketo-azo forms A. The absence of bands attributable to C = N and enolic -OH supported the existence of dyes **1–18** in tautomer form A in the solid state, as shown in Fig. 1. Additionally, the FT-IR spectra showed peaks at ʋ 3094 –3031 cm^− 1^ assigned to aromatic CH, while aliphatic CH peaks in dyes **2–4** and **13–17** appear at ʋ 2930–2835 cm^− 1^.

The data obtained from^1^H NMR spectra of disperse azo dyes **1–18** showed the presence of exchangeable broad singlet signal in the downfield region at δ 12.22–11.36 ppm was assigned to two NH protons at the 1-position and 3-position of the barbituric and thiobarbaturic acid moieties. The more deshielded proton in the range δ 14.27–13.96 ppm was attributed to OH proton. This higher δ value for the OH proton signal is due to hydrogen bonding. The absence of a signal assigned to the methine proton (CH) indicated that disperse azo dyes **1–18** exist in the diketo azo-enol tautomer B in deuterated dimethyl sulfoxide solution. The^1^H NMR of dyes **1–18** exhibited signals in the range δ 8.23–7.04 ppm assigned to aromatic protons of phenyl moieties. Furthermore, dyes **3** and **13–15** showed singlet signals at δ 2.23–3.82 ppm, attributable to methyl groups.

#### Preparation of 1,3-dibenzyl-5-(arylazo)-birbituric acids 19–23

The presence of more than one nucleophilic centre in dyes **1**,** 2**,** 4**,** 6**, **10** encourage us to attempt to further functionalization of them by the reaction with benzyl bromide through an S_N_ reaction to give the disperse azo dyes **19–23** (**19**,** X = H; 20**,** X = CH**_**3**_, **21**,** X = OCH**_**3**_; **22**,** X = Cl; 23**,** X = NO**_**2**_), as shown in **Scheme 3**. It was found that nucleophilic substitution occurs through the two nitrogen atoms of the pyrimidine moiety. This is supported by the FT-IR spectra of dibenzyl compounds **19–23** which indicated the disappearance of N-H stretching vibrations confirm the formation of dibenzylation products **19–23**. Also, their^1^H NMR spectra exhibited signals in the range δ 4.59–5.08 ppm, assigned to the methylene protons of benzyl moieties and the absence of the exchangeable signal for the N-H proton indicated that the dibenzylation reaction had taken place.

**Fig. 2:**
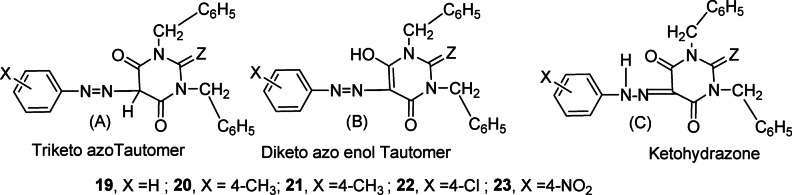
Possible tautomers of 1,3-benzyl-5-(arylazo) barbituric acids **19–23**.

Physical and spectral data of the prepared dyes (**19**,** X = H; 20**,** X = CH**_**3**_, **21**,** X = OCH**_**3**_; **22**,** X = Cl; 23**,** X = NO**_**2**_) are given in the experimental section. They may exist in three tautomeric forms, **A** – **C**, as shown in Fig. 2 in solid or in solution states.

The IR spectra of dibenzyl derivatives **19–23** exhibit three peaks corresponding to the three C = O groups, similar to those found in dyes **1**,** 2**,** 4**,** 5**, and **10**. The peak in the higher region at υ 1727 − 1701 cm^− 1^, is attributed to the stretching vibration of the C = O group at the 2-position. The other two peaks at 1681 − 1673 cm⁻¹ and 1645 − 1630 cm⁻¹ correspond to the stretching vibrations of the C = O groups at the 4- and 6-positions, respectively.“. The absence of NH and OH signals, along with the appearance of a peak at υ 1528 − 1515 cm⁻¹, characteristic of the azo group (-N = N-), supports the presence of tautomer A in the solid state. The frequency results for the various functional groups are consistent with the reported data^25^.

The predominance of the keto-hydrazone form C of the dyes in solution was evidenced by analyzing the ¹H-NMR spectra recorded in DMSO-*d₆*. The enolic -OH signals (> 15 ppm) of the enol forms (Fig. 2, B) are not observed in the spectrum, ruling out the presence of tautomer B in solution. The amide –NH-signals of the keto form (Fig. 2, C) appear around 14.05 − 14.00 ppm, confirming the presence of the tautomer B in solution.

### Electronic absorption spectra of disperse dye 1–23

Electronic absorption provides the most detailed information about the electronic structure and optical properties of molecules. This method is particularly useful for understanding the relationship between dye molecular structure and color, which is crucial for their applications in dyeing of natural and synthetic fibres.

It is well known that the aromatic substituents affect the color intensity of the dyes, due to their ability to increase or decrease the delocalization of electrons through the overall molecule. The absorption spectra of azo dyes are characterized by intense bands in the visible region, typically associated with π→π* and n → π transitions of the azo group and its conjugated system.

**Fig. 3:**
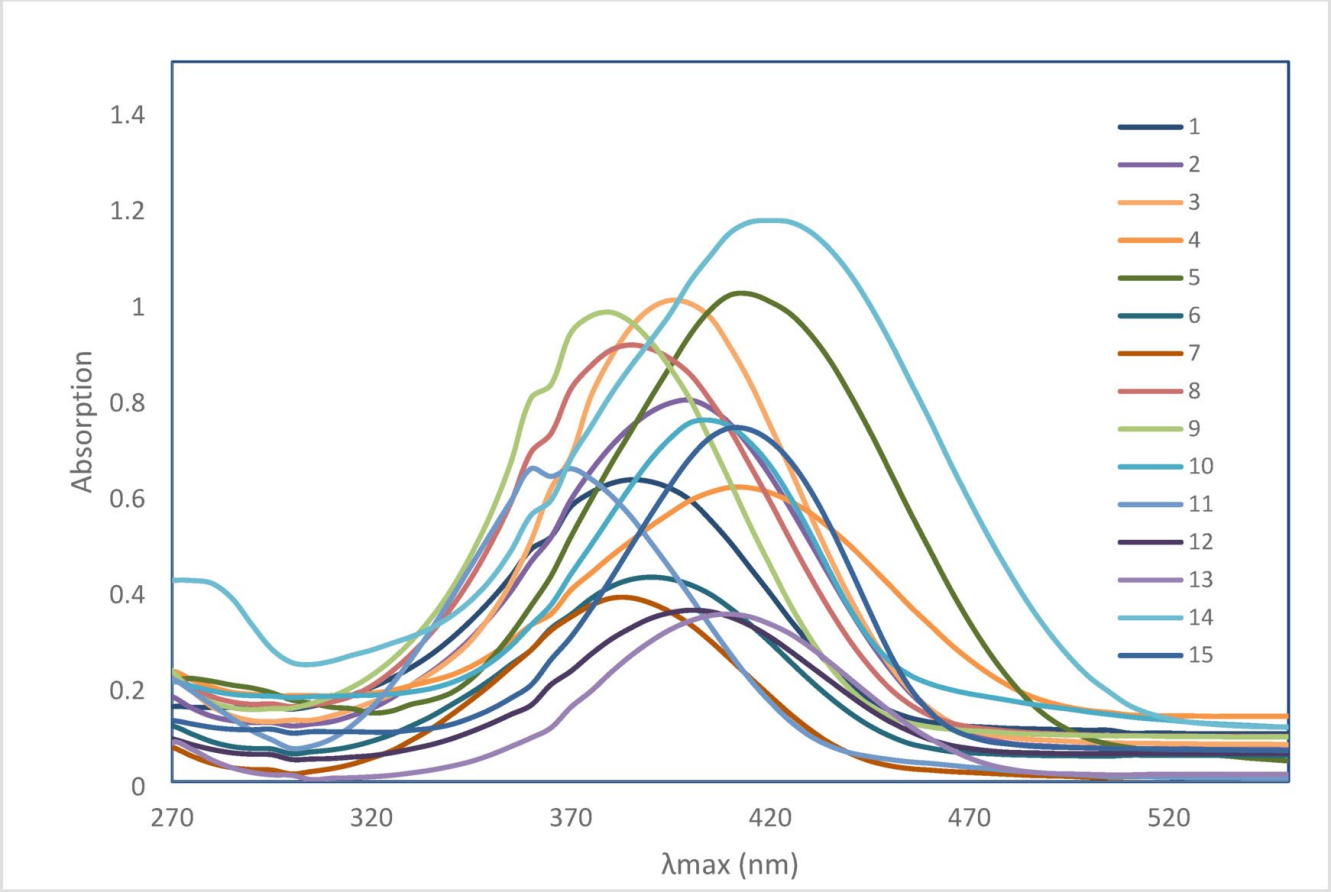
UV spectra scan of dyes **1–15**.

**Fig. 4:**
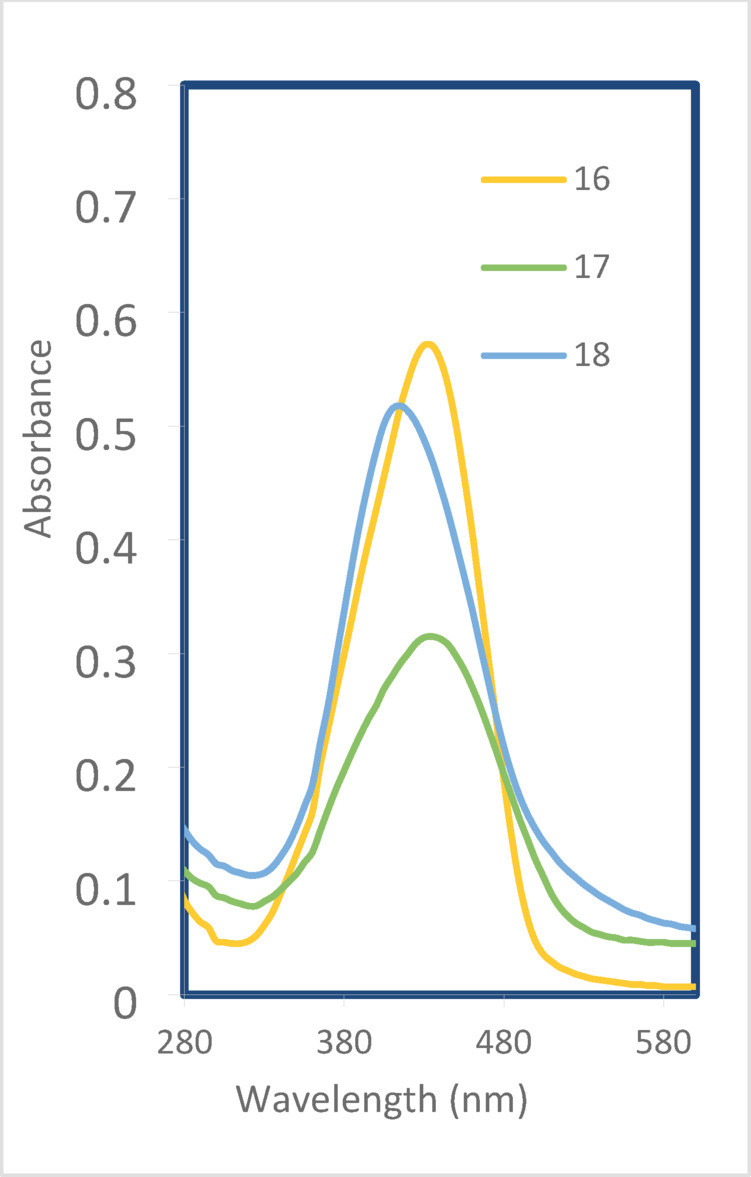
UV spectra scan of dyes **16–18**.

In this study, the experimental and theoretical absorption maxima (*λ*_*max*_) of disperse azo dyes **1–23** were examined in dimethylformamide (DMF). The theoretical calculations were performed using Time-Dependent Density Functional Theory (TD-DFT) with the B3LYP functional and 6-31G(d, p) basis set. A generally good agreement between the experimental and theoretical *λ*_*max*_ values across the series of dyes **1–18** was observed when examining the UV-Vis spectral results presented in Table 1; Figs. 3 and 4. This agreement validates the computational approach and strengthens confidence in using theoretical predictions to determine the electronic properties of these azo dyes.

Trends in *λ*_*max*_ for dyes **1–15 (Z = O)** are notable. Electron-donating groups such as X = methyl (dyes **2** and **3**), X = methoxy (dye **4**) and X = hydroxy (dye **5**) show bathochromic shifts compared to unsubstitued dye 1 (*λ*_*max*_ of 385 nm), with *λ*_*max*_ values of 400 nm, 395 nm, 415 and 415 nm, respectively. This shift can be attributed to the increased electron delocalization through the conjugated system which lowers the energy gap between the ground and excited states resulting in the observed bathochromic shifts.

Table 1 shows that the nature and position of a substituent is presumably affect the UV absorption of these dyes. For example, the strong electron-withdrawing group (X = NO_2_), showed position-dependent effects on the UV wavelength. The 4-nitro derivative (dye **10**) exhibits a bathochromic shift (405 nm), while the 3-nitro derivative (dye **11**) shows a significant hypsochromic shift (360 nm). This difference can be explained due to the extended conjugation in the para position versus the disrupted conjugation in the meta position. Another example of wavelength changes is observed upon the replacement of the sulfur atom in dyes **16–18** (Z = S) instead of the oxygen atom (Z = O) in dyes **1–15**. We observed a consistent bathochromic shift when comparing the azo thiobarbituric acid derivatives (dyes **16–18**) with their azo barbituric acid counterparts. For instance, the 4-methyl derivative (dye **16**,** Z = S**) shows a *λ*_*max*_ of 435 nm compared to 400 nm for its oxygen-containing analog (dye **2**,** Z = O**). This red shift can be attributed to the larger, more polarizable sulfur atom, which enhances the electron-donating ability of the thiobarbituric acid moiety.

**Fig. 5:**
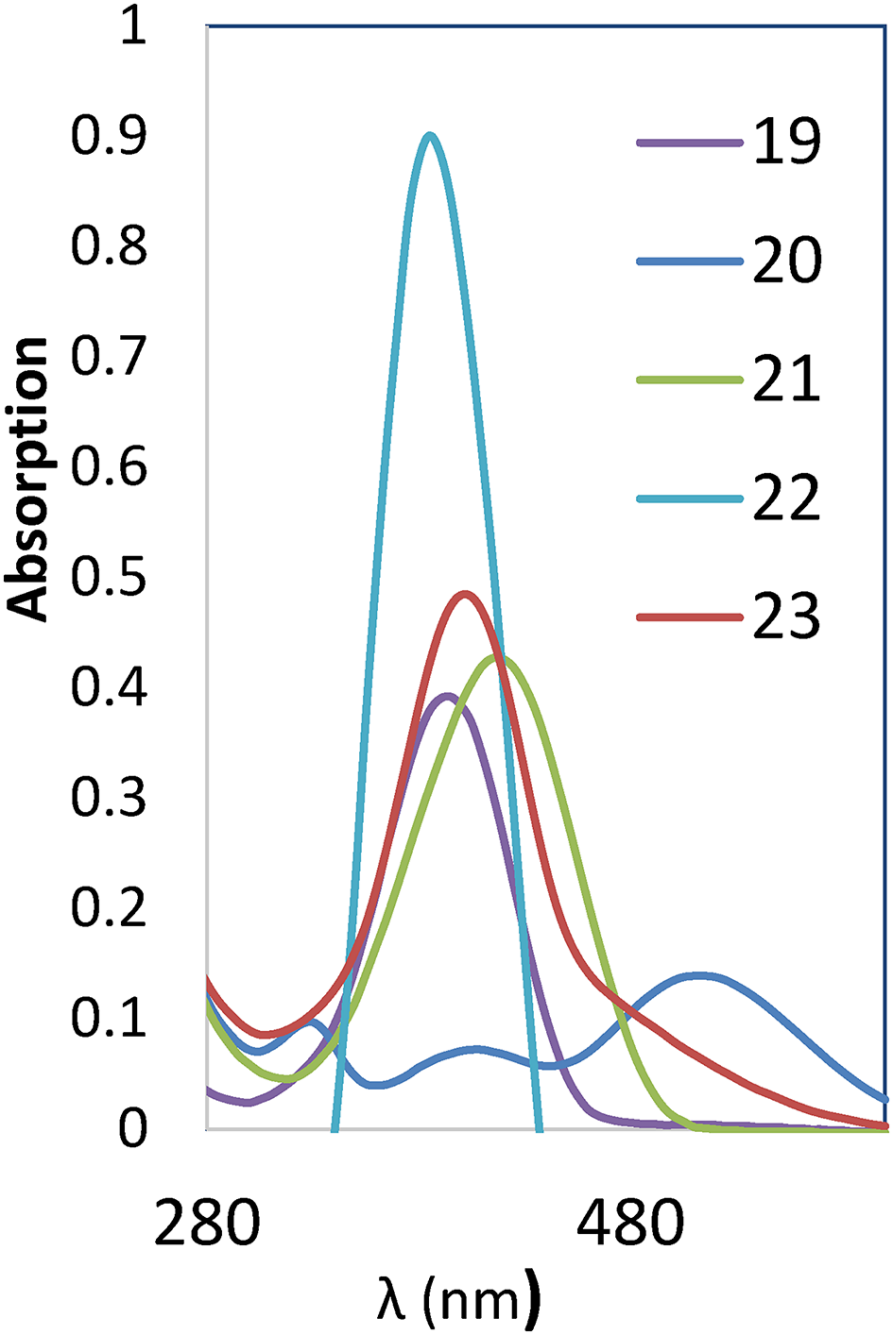
UV spectra scan of dyes **19–23**.

The (*E*)-1,3-dibenzyl-5-(aryldiazenyl)pyrimidine-2,4,6(1*H*,3*H*,5*H*)-trione (dyes **19–23**) generally shows absorption maxima similar to or slightly red-shifted compared to their non-benzylated counterparts, Table 1; Fig. 5. The unsubstituted dye **19** (X = H, *λ*_*max*_ = 395 nm) shows a small bathochromic shift compared to dye **1** (X = H, 385 nm), suggesting that the benzyl groups contribute to extension of the conjugated system. Similar to to dyes **1–18**, there is good agreement between experimental and theoretical results of dyes **19–23**, which validates the computational approach and allows for reliable predictions of optical properties based on molecular structure.

**Table 1:** The experimental and theoretical absorption maxima (*λ*_*max*_) in Nm of disperse Azo dyes **1–23** in DMF, DMF/0.1 M HCl and DMF/0.1 M NaOH.

Dye	X	λ_max_ (DMF) (experimental)	λ_max_ (DMF) (theoretical)	λ_max_ (HCl)	λ_max_ (NaOH)
**1**	4-H	385	371.23	375	395
**2**	4-CH_3_	400	385.80	370	465
**3**	3-CH_3_	395	380.06	370	440
**4**	4-OCH_3_	415	411.27	355	415
**5**	4-OH	415	396.66	340	425
**6**	4-Cl	390	378.71	380	395
**7**	3-Cl	385	361.09	375	405
**8**	4-Br	385	383.37	370	460
**9**	3-Br	380	368.42	370	425
**10**	4-NO_2_	405	387.89	375	415
**11**	3-NO_2_	360	349.69	360	390
**12**	4-SO_3_H	400	380.09	345	405
**13**	3,4-(CH_3_)_2_	410	388.94	370	420
**14**	3,4-(OCH_3_)_2_	420	416.30	390	440
**15**	2-CH_3_-4-NO_2_	410	390.55	395	415
**16**	4-CH_3_	435	417.83	370	440
**17**	4-OCH_3_	440	444.65	360	450
**18**	4-NO_2_	415	420.05	380	420
**19**	-H	395	378.75	395	395
**20**	4-CH_3_	415	424.57	400	420
**21**	4-OCH_3_	415	425.09	410	420
**22**	3-Cl	385	381.98	380	400
**23**	4-NO_2_	405	411.9	400	420

### The effects of acid and base on the absorption maxima of dyes 1–19

The study of acid-base effects on the electronic absorption spectra of azo dyes provides valuable insights into their molecular structure, electronic properties, and potential applications as pH indicators^26–29^. Azo dyes can undergo protonation in acidic conditions, or deprotonation in basic conditions, leading to significant changes in their electronic structure and, consequently, their absorption spectra. These spectral shifts are crucial for understanding the behavior of these dyes in different pH environments and for the applications of these dyesin various fields, including textiles, pH sensing, and analytical chemistry.

We examine the absorption spectra of disperse azo dyes **1–18** in DMF under neutral conditions, as well as after the addition of 0.1 M HCl (acidic conditions, pH = 1) and 0.1 M NaOH (basic conditions, pH = 13). The effects of acid and base on the absorption maxima of the dyes were investigated; and the results are listed in Table 1. Most dyes with either Z = O or S, exhibit hypsochromic shift (blue shift) compared to their neutral state when hydrochloric acid (pH = 1) is added to a dye solution in DMF. This shift is particularly pronounced for compounds with electron-donating groups. For example, dye **4** (X = 4-OCH_3,_ Z = O) shows a significant blue shift from 415 nm to 355 nm, and dye **5** (X = 4-OH, Z = O) shifts from 415 nm to 340 nm. This hypsochromic shift can be attributed to the protonation of the azo group and otherelectronegative atoms, which reduces the electron-donating ability of these substituents and decreases the overall conjugation of the system.

In basic conditions, the disperse azo dyes **1–18** display a bathochromic shift (red shift) compared to their neutral phase. Dye **2** (X = 4-CH_3,_ Z = O) shows a substantial red shift (bathochromic shift) from 400 nm to 465 nm, and dye **8** (X = 4-Br, Z = O) shifts from 385 nm to 460 nm. This behavior is due to: (i) the enhanced electron-donating ability of these groups in basic conditions, increases the overall conjugation of the system, and (ii) transformation of the neutral species of the compound to the anionic species as a result of deprotonation process of any acidic hydrogen atom. Such factors lead toan easier delocalization of electrons, which leads to the observed red shift in *λ*_*max*_ of the band in solution of high pH’s^30^. However, the absorption of both dyes **10** ( X = 4-NO_2_, Z = O) and **18** (X = NO_2_, Z = S) at high pH display another band or shoulder in the wavelength of 415 nm due to the quinone hydrazone tautomer^31^.

The observed pH-dependent spectral changes demonstrate the potential of these azo dyes as promising pH-sensing materials. Compounds like dyes **2**,** 4**,** 5**, and **8** exhibit remarkable chromogenic responses with distinct color changes across different pH ranges, suggesting their applicability in developing colorimetric pH sensors. Notably, the significant bathochromic and hypsochromic shifts, ranging from 60 to 75 nm, indicate high sensitivity to pH variations. These properties align with recent advancements in smart sensing technologies, where responsive dyes can be utilized in various applications such as environmental monitoring, biomedical diagnostics, and intelligent indicator systems^26–29^.

### Dyeing process and Dye-fabric interaction

Disperse dyeing of polyester was carried out using the high temperature (HT) method in Rossari Labtech Flexi Dyer dyeing machine with a material to liquor ratio of 1:20. 2% Dye was used for dyeing (calculated on weight of the fabric). All synthesized azo disperse dyes **1–23** showed less solubility in water. Fine dispersion of the dye in water was obtained after the addition of DYEWELL-002 as a dispersing agent.

**Fig. 6:**
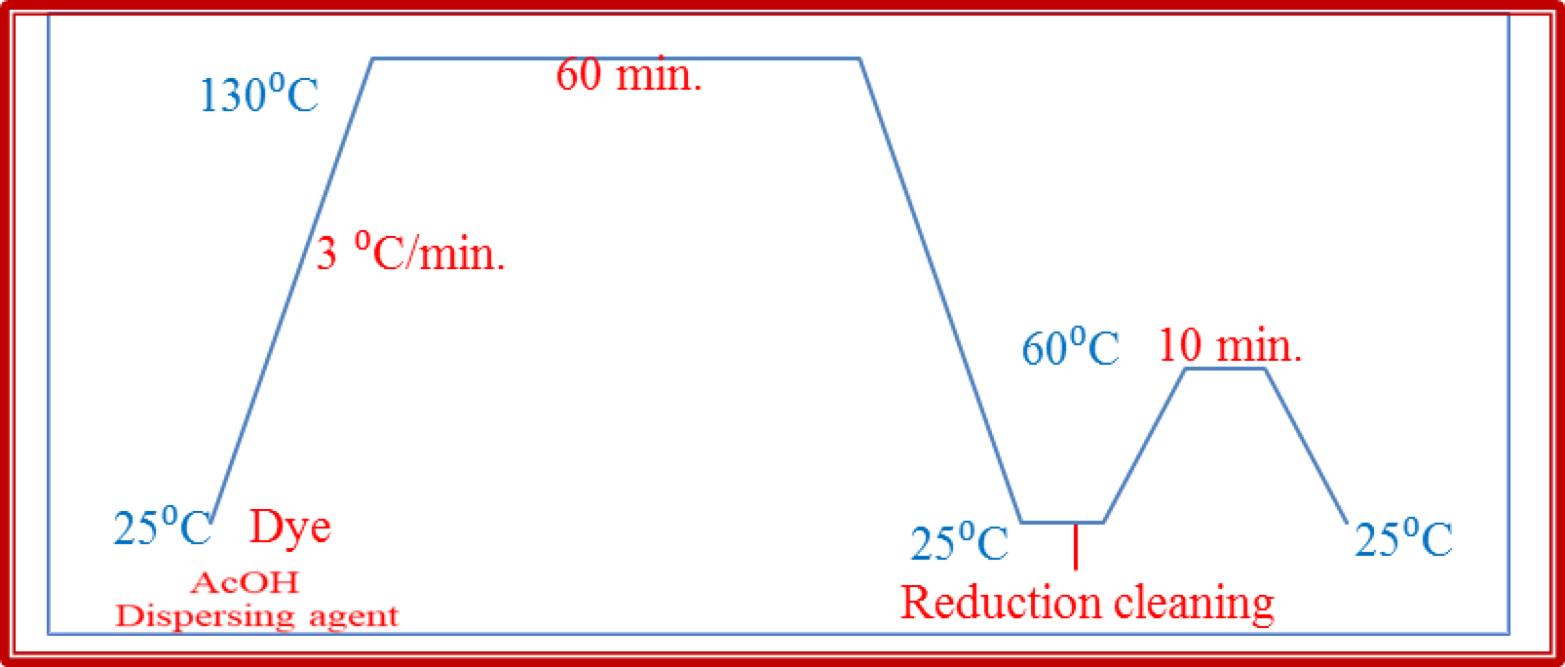
One bath dyeing process of polyester fabrics with dispersed dyes **1–23**.

Under optimal dyeing conditions, the dyebath is adjusted at pH range of 4–5 with acetic acid. This adjustment helps stabilize the disperse dyes environmentally and promotes their interaction with the polyester fibres. The dye bath temperature was raised at a rate of 3 ^o^C/ min to 130 ^o^C, maintained at this temperature for 60 min, and then rapidly cooled to room temperature as shown in Fig. 6. The high dyeing temperature of 130 °C is crucial because it causes the polyester fibres to swell, which creates spaces within the polymer structure. This swelling allows the small, non-ionic dye molecules to diffuse into the fibre. The dyed fabrics were rinsed in cold water and then reduction-cleared in an aqueous solution of 1 g/L sodium hydrosulfite and 1 g/L sodium hydroxide, using a 1:50 liquor-to-goods ratio at 80 °C for 30 min. he reduction step is crucial for removing any loosely bound or unfixed dye molecules from the fabric surface, thereby improving wash fastness and enhancing the overall color quality. The dyed fabrics were washed in cold water and allowed to dry in the open air.

The dispersing agent plays a vital role, enhancing the dye’s solubility and promoting its diffusion into the PE fabric structure. It helps to maintain the dye in a fine dispersion, preventing agglomeration of dye particles, ensuring a uniform distribution throughout the dyebath, preventing crystallization of the dye, and maintaining dispersion stability. This fine dispersion increases the surface area of the dye particles, facilitating their transfer from the aqueous phase into the polyester fibres.

It should be mentioned that the addition of a dispersing agent in the dyeing process did not show a remarkable reduction of the UV-visible absorption intensity of the longest wavelength absorption band of the dye formed between barbituric or thiobarbituric acids and diazotized substituted anilines.

**Fig. 7:**
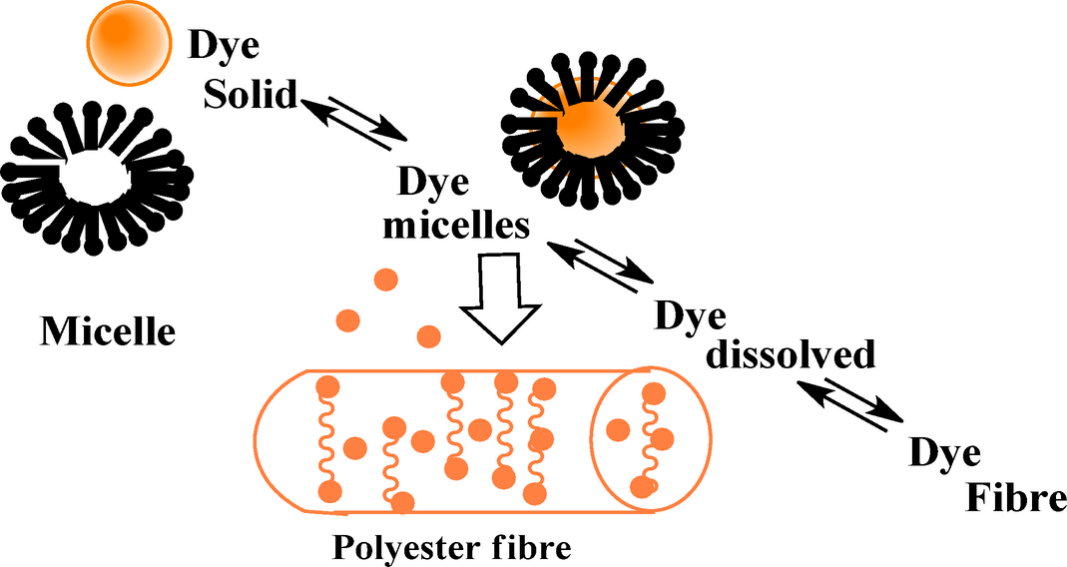
Sketch shows the rule of dispersing agent in dyeing process of polyester.

The inclusion of a dispersing agent in the dyebath is a crucial factor in the application of disperses dyes. Once such a compound is added to water, its dual character results in the formation of micelles at a critical but low concentration. The hydrophobic tails of the dispersing agent molecules are inside the micelle, which allows it to solubilize the disperse dye molecules, thereby conferring higher apparent solubility on the dye. The dye transfers to the fiber from the micelles. As micelles empty their dye, they re-form and dissolve more dye from the solid particles, as shown in Fig. 7.

Once inside the fibre, the dye molecules are held in place by various intermolecular forces, including Van der Waals forces and hydrogen bonding. The subsequent cooling process after dyeing causes the polyester structure to contract, effectively trapping the dye molecules within the fibre. This physical entrapment, combined with the intermolecular forces, contributes to the excellent wash fastness observed in many of the dyed samples.

### Fastness properties of polyester fabrics dyed with disperse dyes 1–23

The fastness properties of dyed textiles are crucial factors that determine the quality, durability, and commercial viability of the dyeing process.

. In this study, we evaluated the fastness properties of polyester fabrics dyed with a series of novel azo dyes **1–23**, as summarized in Table 2. The fastness properties assessed include wash fastness, perspiration fastness (acidic and alkaline), scorch fastness (on cotton and polyester), and light fastness with the standard technique^32,33^. These properties were evaluated using standard techniques and rated on a scale from 1 (poor) to 5 (excellent), with light fastness rated on a separate scale from 1 to 8 ^34^.

**Table 2:**
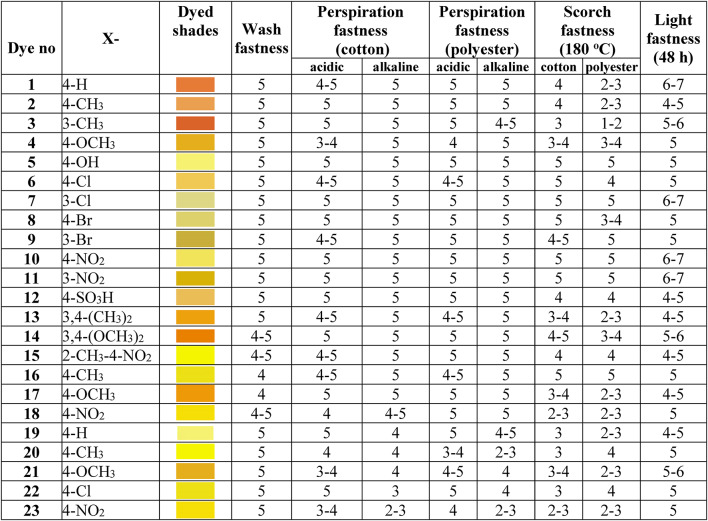
Fastness characteristics of Azo disperse dyes **1–23**.

#### Fastness to washing

Wash fastness depends upon the solubility of dye in water, size of dye molecules, the charge present on the dye, and which type of linkage present in the dye molecule and the fabrics. A sample of dyed fabric was washed in solution (3 g/l Na_2_CO_3_, 1 g/l NaOH) at 60 ^o^C for 30 min. The change in tone of washed fabrics was assessed by the international standard scale IS: 765–1979 (1 for poor and 5 for excellent) given in Table 2. Wash fastness, which measures the resistance of the dyed fabric to color removal during washing, were also found to be in line with or superior to conventional disperse dyes. While standard azo disperse dyes typically exhibit wash fastness ratings of **4–5**, our synthesized dyes **1–23** universally displayed excellent wash fastness (rating = 5). This indicates that the dyes were firmly fixed within the polyester fibres, ensuring minimal color loss during washing processes. The dispersing agent DYEWELL-002 likely contributed to this excellent wash fastness by improving the dye’s penetration and fixation within the fabric.

#### Fastness to light

Light fastness is the degree to which a dye resists fading due to light exposure. All synthesized dyes are quite susceptible to light damage which fully depends on molecular structure. According to the AATCC test method all dye showed very good light fastness on polyester fabrics. Table 2 reveals that dyes **1**,** 7**,** 10**, and **11** exhibited excellent light fastness with ratings of 6–7, indicating their suitability for applications where exposure to sunlight or artificial light is expected.

#### Perspiration fastness

This property assesses the resistance of the dyed fabric to color change or staining caused by perspiration, was evaluated under both acidic and alkaline conditions. Most dyes exhibited an excellent fastness rating of 5 in both acidic and alkaline environments. However, azo dibenzylbarbituric acid **19–23** showed slightly lower perspiration compared to other dyes.

#### Scorch fastness

It measures the resistance of the dyed fabric to color change or damage caused by exposure to high temperatures, showed more variation among the dyes. On cotton, azo barbituric dyes **1–15** exhibited well to excellent scorch fastness with ratings ranging from 4 to 5 except dyes **3**,** 4** and **13** have lower scorch fastness. However, azo thiobarbaturic **16–18** and azo dibenzyl barbituric **19–23** showed moderate scorch fastness. On polyester, the scorch fastness results were more diverse. Dyes **5**,** 7**,** 9**,** 10**, and **11** demonstrated excellent scorch fastness (rating 5) on polyester, while others showed moderate to good performance. This variation in scorch fastness could be attributed to differences in the thermal stability of the dye molecules or their interaction with the polyester fibres at high temperatures.

When comparing the overall performance of the dyes, it is evident that some dyes, such as **5**,** 7**,** 10**, and **11**, consistently demonstrated excellent fastness properties across all tests. These dyes could be considered superior choices for applications requiring high durability and color retention.

### Dye exhaustion and color strength

The study of dye exhaustion and color strength is crucial in textile dyeing processes, particularly for polyester fabrics. Examining the performance of azo dyes **1–23** in terms of dye exhaustion percentage (%E) and color strength (K/S and K/S_sum_) provides valuable information on dye uptake efficiency and the intensity of coloration achieved on the fabric (see Table 3).

The %E measures the proportion of dye absorbed by the polyester fabric from the dye bath, Eq. (1) ^35^, where C1 and C2 represent the initial and final dye concentrations in the bath, respectively. The concentrations were measured using a UV/visible spectrophotometer (PG Instruments T80+). A higher %E value signifies more effective dye uptake by the fabric.

1 $$\:\%E=\frac{{C}_{1}-{C}_{2}}{{C}_{1}}\text{x}\:100$$

Where C1 is absorbance of dyebath before dyeing and C2 absorbance after dyebath after dyeing.

In view of the structure of dyes **1–23**, it is likely that the dye-fiber exhaustion depends chiefly on two hydrophobic interactions between the hydrophobic of the dye and that of the polyester fiber. Owing to the above fact, the higher magnitude of the thermodynamic values of dyes **1–23** on polyester fabric should be considered as a resultant effect of (i) idealized electrostatic attraction of the dyes operative predominantly for the protonated polyester fibers under acidic condition and of (ii) the idealized hydrophobic attraction operative between the dyes and the fibers.

The dyeing performance of the synthesized azo disperse dyes was systematically evaluated and compared with prior studies on structurally similar dyes. Several studies have reported exhaustion percentages ranging between 30 and 80% for conventional azo disperse dyes used on polyester^16^, whereas in this study, exhaustion values ranged from 43.6 to 92.5%. Notably, dye **3** (3-CH_3_) demonstrated an exceptionally high exhaustion rate of 92.5%, surpassing many previously reported disperse dyes.

The %E of the examined dyes show considerable variation, ranging from 43.6% (dye **16**) to 92.5% (dye **3**), presumably is due to significant differences in the affinity of various dyes for the polyester fabric under the present dyeing conditions. The %E value for dye **16** (43.6%) indicates that this disperse dye did not bind easily with the polyester fabric. Notably, dye **3** (3-CH_3_) demonstrates exceptional exhaustion at 92.5%, suggesting it has a particularly high affinity for the polyester fibres. Other dyes showed high exhaustion include dye **4** (4-OCH_3_) at 88.7%, dye **6** (4-Cl) at 88.3%, and dye **9** (3-Br) at 88.4%. In view of the structure of dyes **1–23**, they contain certain substituents, such as methoxy, chloro, and bromo groups, may enhance the dye’s ability to migrate from the dyebath onto the fabric.

The color intensity of the dyed samples is evaluated using the Kubelka-Munk Eq. (2), expressed as the K/S value, which was measured at λ_max_ using a Hunter Lab DP-9000 Color-Spectrophotometer. The surface color yield K/S was used to explain the amount of dye absorbed on the surface of the fiber.

2 $$K/S\:=\:(1-R)^2/2R$$

Where *R* = decimal fraction of the reflectance of the dyed fabric; *Ro* = decimal fraction of the reflectance of the undyed fabric; *K* = absorption coefficient; *S* = scattering coefficient.

The color strength (K/S) values of our synthesized dyes reached up to 22.00 (dye **18**, 4-NO_2_, Z = S**)**, which is significantly higher than the reported values for similar azo disperse dyes used in polyester dyeing^16^. This enhancement in dye performance is likely due to the strong electron-withdrawing effects of NO₂, which increase conjugation and molecular planarity, thereby improving dye-fiber interactions.

To provide a comprehensive assessment of colour strength across the entire visible spectrum (350–750 nm), the K/S_sum_ value was calculated using Eq. (3). This summation offers an overall representation of the dye’s color strength on the fabric.

3 $$\:K/{S}_{\text{s}\text{u}\text{m}}\:=\:\:\sum\:_{350}^{750}{(\text{K}/\text{S})}_{\lambda\:}$$

In terms of color strength, the K/S values vary significantly among the dyes. Dyes **16–18** that were derived from thiobarbituric acid exhibit the highest K/S values compared to those derived from barbituric acid. The high K/S values of dyes **16–18**, indicate intense coloration and suggest that sulphur atom may contribute to the colour development on polyester. Table 3 shows that dye **20** (4-CH_3_) has low K/S value of 1.66, indicating weak color strength,

The K/S_sum_ values, which represent the overall color strength across the visible spectrum, show considerable variation. Dye **17** (4-OCH_3,_ Z = O) has the highest K/S_sum_ of 349.05, followed by dye **18** (4-NO_2_, Z = S) at 339.09 and dye **16** (4-CH_3_, Z = S) at 292.36. These high values suggest that these dyes produce a more intense and broader color profile on the polyester fabric.

The position of substituents on the dye molecule also appears to play a role in both exhaustion and color strength. For example, dye **3** (3-CH_3_, Z = O) has meta-substituted methyl group, showing higher exhaustion and K/S values compared to dye **2** (4-CH3, Z = O) that contain para-substituted methyl group. Similarly, the position of chloro and nitro groups (compare dyes **6** vs. **7** and **10** vs. **11**, Z = O) affects both exhaustion and color strength, where para-substitution generally showing higher K/S_sum_ values. The nature and position of substituents X significantly influence both the dye’s affinity for the fabric and its ability to impart color. These findings can guide the selection and design of disperse dyes for polyester dyeing, helping to optimize both process efficiency and color quality in textile applications.

**Table 3:** Color strength (K/S) value at maximum wavelength, K/S_sum_ and exhaustion percentage (%E) values of dyed polyester dyed by Azo dye **1–23**.

Dyed fabric	X	K/S	K/S_sum_	Dye exhaustion %
**1**	4-H	5.30	56.22	69.0
**2**	4-CH_3_	7.30	262.04	65.8
**3**	3-CH_3_	10.90	123.45	92.5
**4**	4-OCH_3_	2.60	48.90	88.7
**5**	4-OH	5.43	55.70	65.5
**6**	4-Cl	4.36	46.62	88.3
**7**	3-Cl	3.70	33.89	71.5
**8**	4-Br	2.73	30.68	58.2
**9**	3-Br	3.36	29.71	88.4
**10**	4-NO_2_	12.90	116.06	82.0
**11**	3-NO_2_	3.67	28.31	60.0
**12**	4-SO_3_H	5.13	56.40	64.0
**13**	3,4-(CH_3_)_2_	5.53	69.36	70.9
**14**	3,4-(OCH_3_)_2_	2.65	71.42	65.8
**15**	2-CH_3_-4-NO_2_	13.50	136.66	86.9
**16**	4-CH_3_, Z = S	14.50	292.36	43.6
**17**	4-OCH_3 ,_ Z = S	21.40	349.05	56.3
**18**	4-NO_2 ,_ Z = S	22.00	339.09	85.8
**19**	-H	12.10	125.51	53.0
**20**	4-CH_3_	1.66	21.89	68.0
**21**	4-OCH_3_	4.41	80.79	66.0
**22**	4-Cl	16.10	166.76	72.0
**23**	4-NO_2_	9.50	90.82	65.0

### Color measurement

Color measurement is a critical aspect of textile dyeing, providing quantitative data for evaluating and comparing the color properties of dyed fabrics^36^. The CIE L*a*b*color space, widely adopted in the textile industry, offers a three-dimensional representation of color based on human perception. This study examines the colorimetric properties of polyester fabrics dyed with azo dyes **1–23**, measured in the CIE L*a*b* color space using a Hunter Lab DP-9000 Color-Spectrophotometer.

**Fig. 8:**
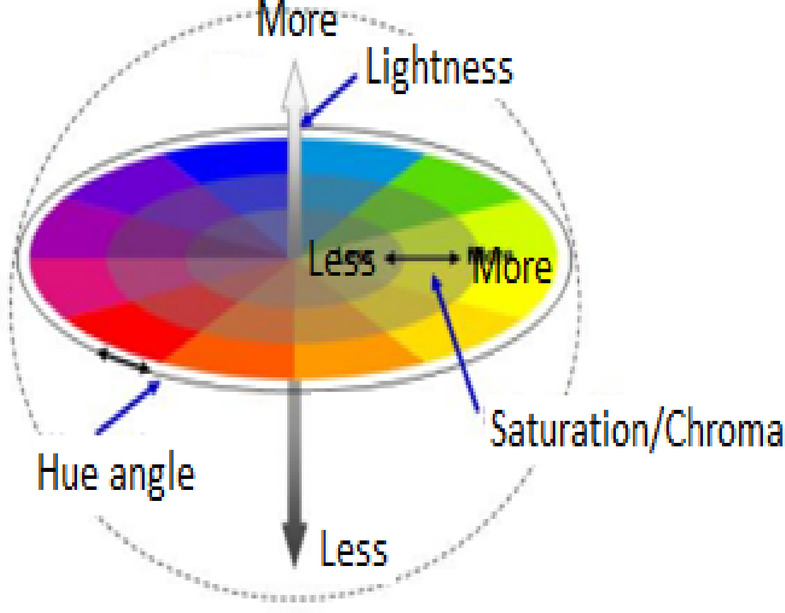
Representation of color coordinate.

In the CIE L*a*b*color space, L* represents the lightness of a color, ranging from 0 (black) to 100 (white). The a* value indicates the red-green axis, with positive values showing redness and negative values showing greenness, while the b* value represents the yellow-blue axis, with positive values indicating yellowness and negative values indicating blueness. The LCh* color space uses the same diagram but with cylindrical coordinates rather than the rectangular coordinates, with L* representing lightness, C* the chroma value, and h^o^ the hue angle. C* (Chroma) describes the saturation or intensity of a color. The chroma values start from 0 in the center to 10, where a higher number corresponds to a color with higher saturation (i.e., black, grey, and white have zero chroma), while hue as an angle represents the hue of the color in degrees. It starts at 0 degrees for red, passes through 90° for yellow, 180° for green and 270° for blue, Fig. 8.

Color differences between dyed fabrics and a reference sample are quantified using ΔL* (difference in lightness), ΔC* (difference in chroma), ΔH* (difference in hue), and ΔE (total color difference), Eq. 4. These parameters collectively provide a comprehensive description of color properties and differences in dyed fabrics.

4 $$\:\Delta\text{E}\text{}\text{=}\text{}\left[\right(\varDelta\:{L}^{*}{)}^{2}+(\varDelta\:{a}^{*}{)}^{2}+(\varDelta\:{b}^{*}{)}^{2}{]}^{\frac{1}{2}}$$

The L* values for the dyed fabrics range from 74.15 (dye **3**, 3-CH_3_, Z = O) to 87.20 (dye **9**, 3-Br, Z = O), indicating a variety of lightness levels depending on the nature of the substituents, Table 4. Dyes **2** (X = 4-CH_3_, Z = O) and **3** (X = 3-CH_3_, Z = O) exhibit the lowest L* values, suggesting darker shades, while dyes **7** ( X = 3-Cl, Z = O) and **9** ( X = 3-Br, Z = O ) showed lighter colors on the polyester fabrics. Regarding a* values, most dyes showed negative values, indicating a shift towards greenness. Accordingly, dye **12** (4-SO_3_H) exhibits the most pronounced green axis (a* = -14.04). However, some dyes, such as **2** (4-CH_3_), **3** (3-CH_3_), **14** (3,4-(OCH_3_)2) and **23** (4-NO_2_), showed positive a* values, suggesting reddish tones. All dyes exhibit positive b* values, indicating a shift towards yellowness. Dye **2** (4-CH_3_) showed the highest b* value of 89.21, suggesting a strong yellow component, whereas dye **11** (3-NO_2_) has the lowest b* value of 18.14, indicating a less pronounced yellow hue. For dye **1** (X = H, Z = O), the a* (green axis) and b* (yellow axis) values support that this dyed fibre is located in the yellowish-green region. For dye **3** (X = 3-CH_3_, Z = O), the values of a* (red) and b* (yellow), the h^o^ value supports that this dyed fibre located in the reddish-yellow (orange) region. The dyed fibre of **13** (X = 3,4-dimethyl, Z = O), is located in the yellowish –green region which agrees with the values of a* and b*. The dyed fibre by dye **16** (X = 4-CH_3_, Z = S) gave a* (green) and b* (yellow) corroborates while the value of its h^o^ and the colour is in the yellowish-green region. Finally, for dyed fiber **20** (1,3-dibenzyl, X = 4-CH3, Z = O), the h^o^ value indicates that the colored fiber is located in the yellowish-green region, aligning with the a* (green) and b* (yellow) values, Table 4.

The C* values, which represent color saturation, vary significantly. Dye **2** (4-CH_3_) exhibits the highest C* value of 90.53, indicating intense color saturation, while dye **11** (3-NO_2_) shows the lowest C* value of 19.05, suggesting a more muted color. Hue angles (h^o^) range from 80.20° (dyes **2** and **23**) to 111.44° (dye **9**), indicating variations in the perceived hues of the dyed fabrics.

To evaluate color differences, dye **1** (4-H) was used as a reference for dyes **2–18**, and dye **19** (-H) for dyes **20–23**. The ΔE values provide insight into the total color difference from the reference. Dye **2** (4-CH_3_) shows the largest ΔE of 49.23 compared to dye **1**, indicating a significant color difference. Dye 2 (4-CH3) exhibits the highest C* value of 90.53, indicating intense color saturation, while dye 11 (3-NO2) shows the lowest C* value of 19.05, suggesting a more muted color. Dye 2 (4-CH3) shows the largest ΔE of 49.23 compared to dye 1. These findings highlight the significant impact of electron-donating substituents, such as the methyl group, particularly when positioned at the 4th position of the benzene ring. The electron-donating nature of the 4-CH_3_ group enhances the electronic system within the chromophore, resulting in stronger color expression. In contrast, dye **6** (4-Cl) exhibits the smallest ΔE of 2.71, suggesting a closer color match to the reference, Table 4.

The position and nature of substituents on the dye molecules appear to play crucial roles in determining the final color properties. For instance, the para-substituted methyl group in dye **2** leads to a more intense yellow color than the meta-substituted methyl group in dye **3**. Similarly, the presence of electron-withdrawing groups like NO_2_ (dyes **10**,** 11**,** 18**) tends to result in darker shades with lower b* values compared to electron-donating groups like OCH_3_ (dyes **4**,** 17**,** 21**), Table 4.

**Table 4:** Colorimetric data CIE lab and CIE LCh color space of dyes **1–23**.

code	X	L*	a*	b*	c*	h^o^	ΔL*	ΔC*	ΔH*	ΔE
**1**	4-H	81.09	-1.86	40.22	40.26	92.65	---	----	---	----
**2**	4-CH_3_	75.68	15.40	89.21	90.53	80.20	-5.41	50.27	-12.45	49.23
**3**	3-CH_3_	74.15	6.00	52.51	52.85	83.48	-6.94	12.59	-9.17	12.36
**4**	4-OCH_3_	84.71	-10,11	56.99	57.88	100.06	3.62	17.62	7.41	16.49
**5**	4-OH	84.23	-8.65	39.84	40.77	102.25	3.14	0.51	9.6	4.51
**6**	4-Cl	83.05	-4.30	37.33	37.58	96.58	1.96	-2.68	3.93	2.71
**7**	3-Cl	87.13	-9.26	27.73	29.24	108.47	6.04	-11.02	15.82	12.38
**8**	4-Br	84.94	-6.39	32.83	33.45	101.02	3.85	-6.81	8.37	7.29
**9**	3-Br	87.20	-9.33	23.76	25.52	111.44	6.11	-14.74	18.79	16.37
**10**	4-NO_2_	86.98	-6.38	22.53	23.42	105.81	5.89	-16.84	13.16	17.76
**11**	3-NO_2_	86.54	-5.83	18.14	19.05	107.82	5.45	-21.12	15.17	22.15
**12**	4-SO_3_H	86.94	-14.04	45.47	47.59	107.15	5.85	7.33	14.5	3.86
**13**	3,4-(CH_3_)_2_	85.71	-12.44	53.77	55.19	103.03	4.62	14.93	10.38	13.10
**14**	3,4-(OCH_3_)_2_	79.81	4.33	64.40	64.54	86.15	-4.28	24.28	-6.5	24.25
**15**	2-CH_3_-4-NO_2_	83.84	-8.55	53.39	54.07	99.10	2.75	13.81	6.45	12.86
**16**	4-CH_3_	82.50	-6.06	92.81	93.01	93.73	1.41	52.75	1.08	52.53
**17**	4-OCH_3_	78.55	-3.46	78.88	93.01	93.73	-2.54	52.75	1.08	38.55
**18**	4-NO_2_	78.57	-3.38	78.95	79.03	92.45	-2.52	52.75	-0.5	38.62
**19**	-H	85.41	-7.39	59.70	60.15	97.06	---	---	--	----
**20**	4-CH_3_	87.06	-6.24	33.85	34.42	100.45	1.65	-25.73	3.39	25.95
**21**	4-OCH_3_	83.35	-3.32	67.46	67.54	92.81	-2.06	7.39	-4.25	8.01
**22**	3-Cl	85.15	-6.35	68.94	69.23	95.27	-0.26	8.34	-1.79	9.32
**23**	4-NO_2_	76.69	7.42	42.98	43.62	80.20	-8.72	-16.53	-16.86	17.08

∆L*, ∆a*, ∆b*, ∆C*, ∆H* for dyes **2–18** calculated with respect to **1** as reference and for dyes **20–23** calculated with respect to **19** as reference.

### DFT study

Density Functional Theory (DFT) calculations have become an invaluable tool in predicting and understanding the electronic properties and reactivity of organic dyes^37–39^. This study employs DFT calculations to investigate the molecular descriptors of azo dyes **1–23**, providing information on their dyeing behaviour and performance on polyester fabrics. The DFT calculations were performed using the B3LYP functional with the 6-31G(d, p) basis set. This computational approach was selected based on its well-established reliability for organic chromophores and azo compounds. The B3LYP hybrid functional provides a good balance between computational efficiency and accuracy for predicting electronic properties of conjugated systems. The 6-31G(d, p) basis set includes polarization functions on both heavy atoms and hydrogen atoms, which is particularly important for accurately modeling the π-conjugated systems and hydrogen bonding interactions present in our dye molecules. This methodology has been successfully employed in previous studies of similar azo disperse dyes and has demonstrated good correlation with experimental spectroscopic data. The analysis focuses on various electronic parameters, including frontier molecular orbitals, ionization potential, electron affinity, and other reactivity indices (see Table 5), which collectively contribute to the dyes’ interactions with polyester fibres^40–43^.

To validate our computational approach and assess its accuracy, we benchmark our results against prior computational studies, particularly the DFT analysis of barbituric acid-based chalcone disperse dyes^16^.

The frontier molecular orbitals, represented by the Highest Occupied Molecular Orbital (HOMO) and the Lowest Unoccupied Molecular Orbital (LUMO), play a crucial role in determining the electronic properties and chemical reactivity of dye molecules^44^. The HOMO energy indicates the molecule’s electron-donating ability, while the LUMO energy represents its electron-accepting capability. The energy gap (ΔE) between HOMO and LUMO levels is a key indicator of molecular reactivity and potential dye-fibre interactions^45^.

In the referenced study^16^, the HOMO-LUMO gap (ΔE) for barbituric acid-based chalcone dyes was found to range between 0.10 and 0.20 eV, with lower energy gaps correlating to increased dyeing efficiency and stronger dye-fiber interactions. Our computed ΔE values (ranging from 0.1177 eV to 0.2197 eV) fall within this range, further supporting the observed relationship between electronic properties and experimental dyeing performance. Among the studied dyes, dye **17** (X = 4-OCH_3_, Z = S) exhibits the smallest energy gap (ΔE = 0.1177 eV), suggesting potentially higher reactivity and stronger interactions with polyester fibres. This theoretical prediction is strongly supported by experimental results, where dye **17** demonstrated the highest K/S and K/Ssum values (21.40 and 349.05, respectively). The small HOMO-LUMO gap facilitates electronic transitions, enhancing the dye’s ability to absorb visible light and improving its color strength. Additionally, the reduced gap indicates greater molecular softness, which promotes stronger van der Waals interactions with polyester chains, leading to improved dye uptake and exhaustion. Conversely, dye **3** (X = 3-CH_3_, Z = O) showed the largest energy gap (ΔE = 0.21967 eV), indicating lower reactivity and weaker fiber interactions that reduces dyeing performance.

**Fig. 9:**
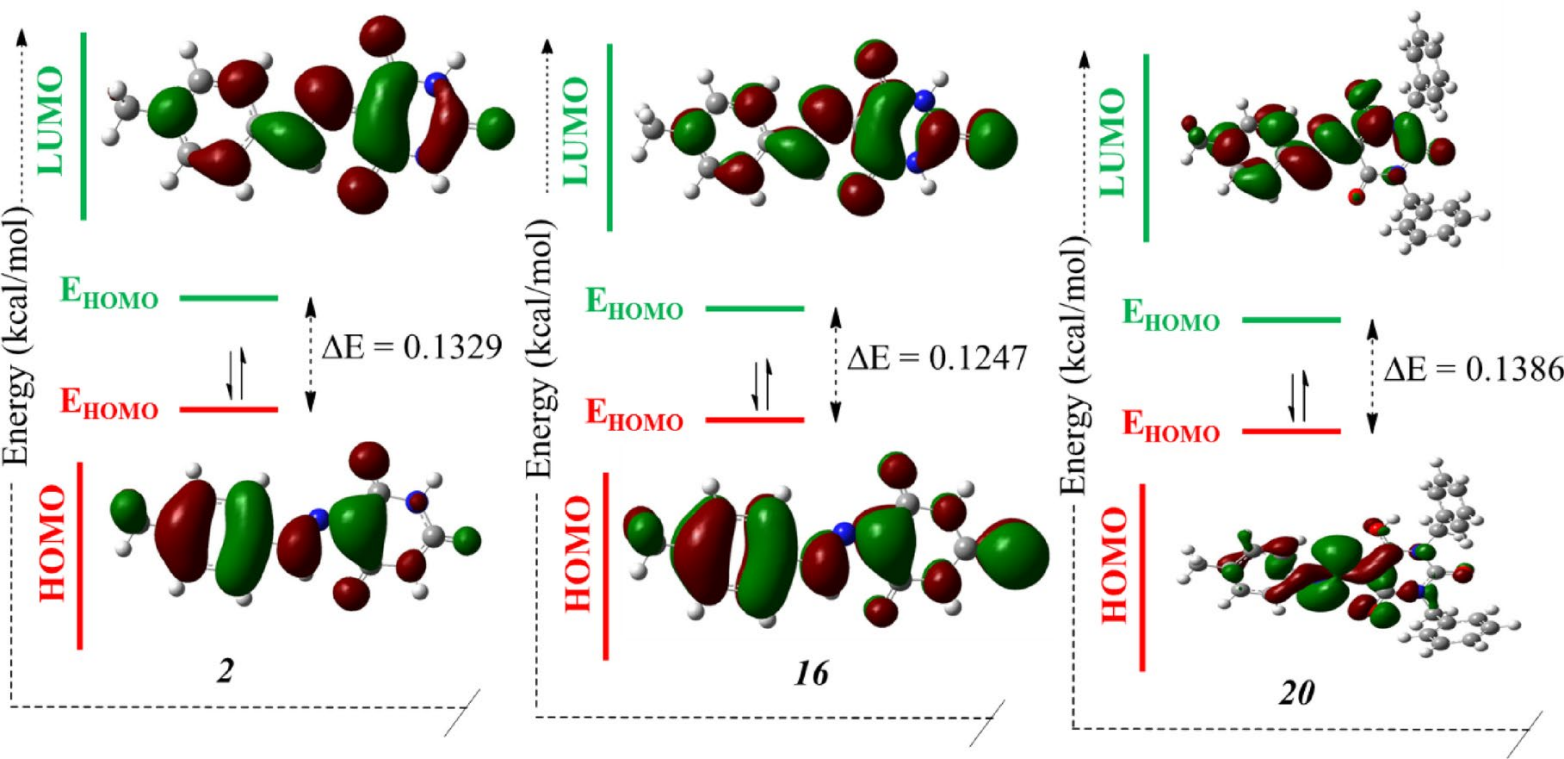
HOMO, LUMO and energy gap of disperse azo dyes **2**,** 16** and **20**.

The variation in energy gaps across the dyes suggests that substituents significantly influence the electronic structure and, consequently, the dyeing behaviour of these compounds. Figure 9 shows the HOMO and LUMO energy diagram of a prototype azo dyes which demonstrates that compounds **3–6** (**3**, X = 3-CH_3_, Z = O; **4**, X = 4-OCH_3_, Z = O; **5**, X = 4-OH, Z = O; **6**, X = 4-Cl, Z = O ) exhibit similar electron cloud distribution patterns with electron clouds distributed mainly over the phenyl ring for the HOMO orbital and over the pyrimidine ring for LUMO orbital.

**Table 5:** Chemical descriptor parameters of dyes **1–23**.

Dye	E_HOMO_ (eV)	E_LUMO_ (eV)	∆E (eV)	IP (eV)	EA (eV)	χ (eV)	µ (eV)	η (eV)	σ (eV^− 1^)	ω (eV)	µ (D)
**1**	-0.2324	-0.0958	0.1366	0.2327	0.0958	0.1641	-0.1641	0.0683	14.6467	0.1972	5.7647
**2**	-0.2261	-0.0932	0.1329	0.2261	0.0932	0.1597	-0.1597	0.0665	15.0478	0.1918	7.1807
**3**	-0.2881	-0.0684	0.2197	0.2881	0.0684	0.1783	-0.1783	0.1098	9.1046	0.1446	7.2237
**4**	-0.2151	-0.0898	0.1252	0.2151	0.0898	0.1525	-0.1525	0.0626	15.9693	0.1856	4.4359
**5**	-0.2041	-0.0648	0.1394	0.2041	0.0648	0.1345	-0.1345	0.0697	14.3493	0.1297	8.4436
**6**	-0.2350	-0.1019	0.1331	0.2350	0.1019	0.1685	-0.1685	0.0666	15.0252	0.2132	5.8810
**7**	-0.2267	-0.0845	0.1421	0.2267	0.0845	0.1556	-0.1556	0.0711	14.0726	0.1704	3.8467
**8**	-0.2335	-0.1019	0.1316	0.2335	0.1019	0.1677	-0.1677	0.0658	15.2022	0.2137	3.5772
**9**	-0.2397	-0.1024	0.1374	0.2397	0.1024	0.1710	-0.1711	0.0687	14.5592	0.2130	2.8626
**10**	-0.2283	-0.1026	0.1258	0.2283	0.1026	0.1655	-0.1655	0.0629	15.9046	0.2177	2.4658
**11**	-0.2259	-0.0922	0.1338	0.2259	0.0922	0.1590	-0.1590	0.0669	14.9533	0.1891	4.0962
**12**	-0.2526	-0.1132	0.1395	0.2526	0.1132	0.1829	-0.1829	0.0697	14.3410	0.2399	9.9774
**13**	-0.2235	-0.0917	0.1318	0.2235	0.0917	0.1576	-0.1576	0.0659	15.1745	0.1884	2.5181
**14**	-0.2133	-0.0893	0.1240	0.2133	0.0893	0.1513	-0.1513	0.0620	16.1355	0.1847	1.8678
**15**	-0.2510	-0.1170	0.1340	0.2510	0.1170	0.1841	-0.1840	0.0670	14.9243	0.2527	4.5726
**16**	-0.2288	-0.1042	0.1247	0.2288	0.1042	0.1665	-0.1665	0.0623	16.0424	0.2224	6.5283
**17**	-0.2185	-0.1009	0.1177	0.2185	0.1009	0.1597	-0.1597	0.0588	16.9952	0.2167	5.3356
**18**	-0.2337	-0.1103	0.1234	0.2337	0.1103	0.1720	-0.1720	0.0617	16.21402	0.2399	3.2718
**19**	-0.2015	-0.0630	0.1385	0.2015	0.0630	0.1323	-0.1323	0.0692	14.4425	0.1264	6.1372
**20**	-0.1992	-0.0605	0.1386	0.1992	0.0605	0.1299	-0.1299	0.0693	14.4279	0.1216	5.9982
**21**	-0.1948	-0.0566	0.1381	0.1948	0.0566	0.1257	-0.1257	0.0691	14.4802	0.1144	4.5359
**22**	-0.2078	-0.0701	0.1378	0.2078	0.0701	0.1390	-0.1390	0.0689	14.5180	0.1402	7.2113
**23**	-0.2186	-0.0958	0.1229	0.2186	0.0958	0.1572	-0.1572	0.0614	16.2774	0.2011	10.2488

The Ionization Potential (IP) and Electron Affinity (EA) provide further information on the dyes’ electronic properties^46^. Furthermore, the ionization potential (IP) and electron affinity (EA) values calculated in our study are consistent with the reported range for similar disperse dyes. Dye **3** (X = 3-CH_3_, Z = O) exhibits the highest relative IP value (0.2881 eV), indicating a greater resistance to electron loss. In contrast, dye **21** (4-OCH_3_, Z = O, 1,3- dibenzyl) shows the lowest IP (0.1948 eV), suggesting it may more readily lose electrons. Regarding EA, dye **15** (2-CH_3_-4-NO_2_, Z = O) displays the highest value (0.1170 eV), indicating a strong electron-accepting capability, while dye **21** (X = 4-OCH_3_, Z = O, 1,3-dibenzyl) shows the lowest EA (0.0567 eV). The chemical potential (µ) and electronegativity (χ) provide insights into the overall reactivity of the dye molecules^47^. Dye **15** (X = 2-CH_3_-4-NO_2_, Z = O) possesses the highest χ value (0.1840 eV), suggesting potentially stronger electron-accepting behaviour, while dye **21** (X = 4-OCH_3_, Z = O, 1,3-dibenzyl) has the lowest χ value (0.1257 eV), indicating weaker electron-accepting tendencies.

Global hardness (η) and softness (σ) parameters offer information about the polarizability and reactivity of the dye molecules^48,49^. Dye **17** (X = 4-OCH_3_, Z = S) displays the highest softness value (16.9952 eV^− 1^), indicating its potential for stronger dye-fibre interactions and enhanced dyeing performance.

The electrophilicity index (ω) measures the stabilization energy resulting from the maximum electron transfer between donor and acceptor^50,51^. Dye **15** (2-CH_3_-4-NO_2_, Z = O) exhibits the highest electrophilicity index (0.2527 eV), suggesting its potential for high dye-fibre affinity on polyester fabrics. In contrast, dye **21** (X = 4-OCH_3_, Z = O, 1,3-dibenzyl) shows the lowest ω value (0.1144 eV). The dipole moment (µ) provides insights into the polarity and charge distribution within the dye molecules. Our computed dipole moments (ranging up to 10.24 D for dye 23) closely match the dipole moments reported for barbituric acid-based chalcone dyes in the previous study^16^. The strong correlation between high dipole moments and enhanced dyeing properties is well-documented, as increased polarity enhances intermolecular interactions between the dye and polyester fibers. It’s noteworthy that the position and nature of substituents on the dye molecules significantly influence their electronic properties.

## Conclusion

This comprehensive study of 23 azo disperse dyes based on barbituric and thiobarbituric acid derivatives has provided valuable insights into their synthesis, spectral properties, dyeing performance on polyester fabrics, and electronic characteristics. The investigation revealed significant correlations between molecular structure and dye performance. Notably, the type and position of substituents on the dye molecules played crucial roles in determining their spectral properties, dyeing behavior, and electronic characteristics.

The excellent wash fastness observed across all dyes (a rating 5) demonstrates their strong affinity for polyester fibres. The wide range of dye exhaustion percentages (43.6–92.5%) and color strength values (K/S from 1.66 to 22.00) highlights the impact of molecular structure on dyeing efficiency. The pH-dependent spectral shifts observed suggest potential applications beyond textile dyeing, such as pH indicators.

DFT calculations provide valuable insights into the electronic properties of the dyes, with HOMO-LUMO energy gaps correlating well with experimental color strength values. For instance, dye **17** (4-OCH_3_) exhibits the highest K/S and K/S_sum_ values, which aligned with its low HOMO-LUMO gap and high softness, indicating strong dye-fiber interactions.

The synthesized azo disperse dyes **1–23** offer promising commercial potential due to their excellent wash fastness and tunable color properties. The high exhaustion rates of several dyes in this series could lead to more efficient dyeing processes with reduced effluent treatment costs. Furthermore, the structure-property relationships established in this study provide valuable guidelines for industrial chemists to design dyes with predetermined properties tailored for specific applications. Future research explores the application of these dyes in technical textiles and smart materials, particularly leveraging their pH-responsive properties for developing color-changing fabrics. Further studies on biodegradability and ecotoxicity of these dyes would also be valuable to align with growing industrial sustainability requirements.

## **Experimental**

### General method for synthesis of disperse dyes 1–18

**Step 1: Preparation of diazonium 2**: In a 250 mL capacity conical flask, aniline derivatives (0.03 mol) were added to mixture of 15 mL conc. HC1 and 10 mL cold water and stirred for 15 min. until a clear solution was obtained. The mixture was cooled to 0–5 °C using an ice-salt bath and maintained at this temperature throughout the diazotization process. Sodium nitrite (4.14 g, 0.06 mol) was dissolved in 10 mL cold water (5 °C) and then added dropwise to the aniline solution over a period of 30 min with constant stirring, maintaining the temperature below 5 °C. The diazotization process was continued for an additional 30 min after complete addition of sodium nitrite solution.

**Step 2: Reaction of diazonium salt with** c**oupling reagent**: The coupling components (barbituric acid or thiobarbituric acid, 0.03 mol) were dissolved in 17 mL ethanol (95%) and 30.0 g sodium acetate in 15 mL of water then cooled to 0–5 °C. The diazonium salt solution prepared in Step 1 was added dropwise to the stirred cooled mixture of coupler and sodium acetate solution over a period of 45 min, maintaining the temperature below 5 °C. The reaction mixture was stirred for an additional 3 h at 5–10 °C and then allowed to reach room temperature. The progress of the reaction was monitored by TLC using ethyl acetate: hexane (3:7) as the mobile phase and visualized under UV light (254 nm). The crude dyes were filtered using a Büchner funnel under vacuum, washed with hot water (60 °C, 50 mL) five times to remove inorganic salts, and then dried in a vacuum oven at 60 °C for 12 h.

**(*E*)-5-(phenyldiazenyl)pyrimidine-2**,**4**,**6(1*H***,**3*H***,**5*H*)-trione 1**.

Pale yellow crystals, 4.2 g (80.50%) yield; m.p. 280 ºC. UV: λ_max_ (DMF): 385 nm, and Ɛ_max_ 62,900 mol^− 1^dm^3^cm^− 1^. IR (KBr): ῡ 3257 (N-H), 3170 (N-H), 3094 (SP^2^ = C-H), 1753 (C = O), 1707 (C = O), 1655 (C = O) and 1512 (N = N) cm^− 1^.

**(*E*)-5-(*p*-tolyldiazenyl)pyrimidine-2**,**4**,**6(1*H***,**3*H***,**5*H*)-trione 2**.

Marigold crystals, 4.5 g (86.57%) yield; m.p. 280 ºC. UV: λ_max_ (DMF): 400 nm, and Ɛ_max_ 78,000 mol^− 1^dm^3^cm^− 1^. IR (KBr): ῡ 3413 (N-H), 3255 (N-H), 3086 (SP^2^ = C-H), 2919 (SP^3^-C-H), 1754 (C = O), 1705 (C = O), 1655 (C = O), and 1513 (N = N) cm^− 1^.

**(*E*)-5-(*m*-tolyldiazenyl)pyrimidine-2**,**4**,**6(1*H***,**3*H***,**5*H*)-trione 3**.

Reddish brown crystals, 2.1 g (77.35%) yield; m.p. 250 ºC. UV: λ_max_ (DMF): 395 nm, and Ɛ_max_ 100,300 mol^− 1^dm^3^cm^− 1^ IR (KBr): ῡ 3176 (N-H), 3061 (SP^2^ = C-H), 2930 (Sp^3^ -C-H), 1725 (C = O), 1694 (C = O), 1662 (C = O) and 1534 (N = N) cm^− 11^. H NMR (400 MHz, DMSO): δ 12.05 (s, 2H, 2NH), 7.50–7.29 (m, 3H, Ar-H), 7.04 (s, 1 H, Ar-H) and 2.35 (s, 3H, CH_3_) ppm^13^. C NMR (101 MHz, DMSO): δ 161.86, 150.99, 143.25, 139.59, 129.85, 127.12, 117.98, 117.61, 114.80, 21.48 (CH_3_) ppm. C_11_H_10_N_4_O_3_ requires: C, 53.65; H, 4.06; N, 22.70%. Found: C, 53.56; H, 4.12; N, 22.64%.

**(*E*)-5-((4-methoxyphenyl)diazenyl)pyrimidine-2**,**4**,**6(1*H***,**3*H***,**5*H*)-trione 4**.

Yellow crystals, 3.1 g (74.20%) yield; m.p. 280 ºC. UV: λ_max_ (DMF): 415 nm, and Ɛ_max_ 61,300 mol^− 1^dm^3^cm^− 1^. IR (KBr): ῡ 3245 (N-H), 3170 (N-H), 3079 (SP^2^ = C-H), 2835 (SP^3^-C-H), 1755 (C = O), 1704 (C = O), 1654 (C = O) and 1513 (N = N) cm^− 1^.

**(*E*)-5-((4-hydroxyphenyl)diazenyl)pyrimidine-2**,**4**,**6(1*H***,**3*H***,**5*H*)-trione 5**.

Reddish brown crystals, 1.2 g (68.57%) yield; m.p. 285 ºC. UV: λ_max_ (DMF): 415 nm, and Ɛ_max_ 10,140 mol^− 1^dm^3^cm^− 1^. IR (KBr): ῡ 3370 (N-H), 3121 (N-H), 3031 (SP^2^ = C-H) and 1705 (C = O) cm^− 1^.

**(*E*)-5-((4-chlorophenyl)diazenyl)pyrimidine-2**,**4**,**6(1*H***,**3*H***,**5*H*)-trione 6**.

Yellow crystals, 4.8 g (83.33%) yield; m.p. 280 ºC. UV: λ_max_ (DMF): 470 nm, and Ɛ_max_ 42,100 mol^− 1^dm^3^cm^− 1^. IR (KBr): ῡ 3257 (N-H), 3176 (N-H), 3085 (SP^2^ = C-H), 1755 (C = O), 1706 (C = O), 1654 (C = O) and 1513 (C = N) cm^− 1^.

**(*E*)-5-((3-chlorophenyl)diazenyl)pyrimidine-2**,**4**,**6(1*H***,**3*H***,**5*H*)-trione 7**.

Brown crystals, 2.16 g (75.57%) yield; m.p. 290 ºC (decompose). UV: λ_max_ (DMF): 385 nm, and Ɛ_max_ 38,200 mol^− 1^dm^3^cm^− 1^. IR (KBr): ῡ 3278 (N-H), 3203 (N-H), 3092 (SP^2^ = C-H), 1756 (C = O), 1712 (C = O), 1654 (C = O) and 1519 (N = N) cm^− 1^^1^. H NMR (400 MHz, DMSO): δ 13.96 (s, 1 H, barbituric acid OH ), 11.57 (s, 1 H, barbituric acid NH), 11.36 (s, 1 H, barbituric acid NH ), 7.67 (s, 1 H, Ar-H), 7.56 (d, *J* = 8.3 Hz, 1 H, Ar-H ), 7.47 (t, *J* = 8.0 Hz, 1 H, Ar-H ) and 7.27 (d, *J* = 7.7 Hz, 1 H, Ar-H ) ppm^13^. C NMR (101 MHz, DMSO): δ 162.28 (C = O), 160.22 (C = O), 150.25, 143.47, 134.60, 131.92, 125.78, 119.17, 116.62 and 115.85 ppm. C_10_H_9_ClN_4_O_3_ requires: C, 44.70; H, 3.34; N, 20.85%. Found: C, 44.63; H, 3.39; N, 20.78%.

**(*E*)-5-((4-bromophenyl)diazenyl)pyrimidine-2**,**4**,**6(1*H***,**3*H***,**5*H*)-trione 8**.

Straw crystals, 3.4 g (76.57%) yield; m.p. 280 ºC. UV: λ_max_ (DMF): 385 nm, and Ɛ_max_ 91,000 mol^− 1^dm^3^cm^− 1^. IR (KBr): ῡ 3250 (N-H), 3197 (N-H), 3079 (SP^2^ = C-H), 1755 (C = O), 1705 (C = O), 1655 (C = O) and 1511 (N = N) cm^− 1^.

**(*E*)-5-((3-bromophenyl)diazenyl)pyrimidine-2**,**4**,**6(1*H***,**3*H***,**5*H*)-trione 9**.

Olive crystals, 4.5 g (86.57%) yield; m.p. 280 ºC. UV: λ_max_ (DMF): 380 nm, and Ɛ_max_ 97,000 mol^− 1^dm^3^cm^− 1^. IR (KBr): ῡ 3277 (N-H), 3174 (N-H), 3086 (SP^2^ = C-H), 1755 (C = O), 1709 (C = O), 1654 (C = O) and 1515 (N = N) cm^− 1^.

**(*E*)-5-((4-nitrophenyl)diazenyl)pyrimidine-2**,**4**,**6(1*H***,**3*H***,**5*H*)-trione 10**.

Pale yellow crystals, 2.2 g (68.57%) yield; m.p. 280 ºC. UV: λ_max_ (DMF): 405 nm, and Ɛ_max_ 75,300 mol^− 1^dm^3^cm^− 1^. IR (KBr): ῡ 3464 (N-H), 3206 (N-H), 3066 (SP^2^ = C-H), 1712 (C = O) and 1514 (N = N) cm^− 1^.

**(*E*)-5-((3-nitrophenyl)diazenyl)pyrimidine-2**,**4**,**6(1*H***,**3*H***,**5*H*)-trione 11**.

Gold crystals, 4.3 g (81.57%) yield; m.p. 290 ºC. UV: λ_max_ (DMF): 360 nm, and Ɛ_max_ 65,200 mol^− 1^dm^3^cm^− 1^. IR (KBr): ῡ 3258 (N-H), 3196 (N-H), 3071 (SP^2^ = C-H), 1758 (C = O), 1669 (C = O) and 1516 (N = N) cm^− 1^.

**(*E*)-4-((2**,**4**,**6-trioxohexahydropyrimidin-5-yl)diazenyl)benzenesulfonic acid 12**.

Red crystals, 3.4 g (76.57%) yield; m.p. 280 ºC. UV: λ_max_ (DMF): 400 nm, and Ɛ_max_ 35,200 mol^− 1^dm^3^cm^− 1^. IR (KBr): ῡ 3257 (N-H), 3172 (N-H), 3088 (SP^2^ = C-H), 1755 (C = O), 1705 (C = O), 1655 (C = O) and 1592 (N = N) cm^− 1^.

**(*E*)-5-((3**,**4-dimethylphenyl)diazenyl)pyrimidine-2**,**4**,**6(1*H***,**3*H***,**5*H*)-trione 13**.

Gold crystals, 1.0 g (61.58%) yield; m.p. 230 ºC. UV: λ_max_ (DMF): 410 nm, and Ɛ_max_ 34,900 mol^− 1^dm^3^cm^− 1^. IR (KBr): ῡ 3264 (N-H), 3192 (N-H), 3081 (SP^2^ = C-H), 2907 (Sp^3^ -C-H), 1725 (C = O), 1692 (C = O), 1616 (C = O) and 1532 (N = N) cm^− 1^^1^. H NMR (400 MHz, DMSO): δ 11.39 (s, 3H, barbituric acid NH), 7.37 (s, 1 H, Ar-H), 7.30 (d, *J* = 8.1 Hz, 1 H, Ar-H), 7.22 (d, *J* = 8.2 Hz, 1 H, Ar-H), 2.27 (s, 3H, CH_3_) and 2.23 (s, 3H, CH_3_) ppm^13^. C NMR (101 MHz, DMSO): δ 150.14, 139.48, 138.22, 135.16, 130.94, 117.97, 117.49, 114.76, 19.94 (CH_3_) and 19.41 (CH_3_) ppm. C_12_H_12_N_4_O_3_ requires: C, 55.38; H, 4.61; N, 21.51%. Found: C, 55.42; H, 4.56; N, 21.57%.

**(*E*)-5-((3**,**4-dimethoxyphenyl)diazenyl)pyrimidine-2**,**4**,**6(1*H***,**3*H***,**5*H*)-trione 14**.

Dark brown crystals, 1.0 g (62%) yield; m.p.250 ºC. UV: λ_max_ (DMF): 420 nm, and Ɛ_max_ 116,900 mol^− 1^dm^3^cm^− 1^. IR (KBr): ῡ 3512 (N-H), 3188 (N-H), 3080 (SP^2^ = C-H), 2925 (Sp^3^ -C-H), 1725 (C = O), 1659 (C = O) and 1519 (N = N) cm-^11^H NMR (400 MHz, DMSO): δ 12.22 (s, 3H, barbituric acid NH ), 7.27 (s, 1 H, Ar-H), 7.14 (d, *J* = 8.7 Hz, 1 H, Ar-H) and 7.04 (d, *J* = 8.7 Hz, 1 H, Ar-H), 3.82 (s, 3H, OCH_3_) and 3.79 (s, 3H, OCH_3_) ppm^13^. C NMR (101 MHz, DMSO): δ 161.65 (C = O), 150.44, 150.09, 148.00, 136.06, 116.93, 112.77, 109.66, 101.56, 56.27 (OCH_3_) and 56.14 (OCH_3_) ppm. C_12_H_12_N_4_O_5_ requires: C,49.31; H, 4.10; N, 19.17%. Found: C, 49.37; H, 4.14; N, 19.24%.

**(*E*)-5-((2-methyl-4-nitrophenyl)diazenyl)pyrimidine-2**,**4**,**6(1*H***,**3*H***,**5*H*)-trione 15**.

Brown crystals, 3.0 g (75.38%) yield; m.p. 250 ºC. UV: λ_max_ (DMF): 410 nm, and Ɛ_max_ 73,700 mol^− 1^dm^3^cm^− 1^ IR (KBr): ῡ 3530 (N-H), 3483 (N-H), 3007 (SP^2^ = C-H), 2855 (Sp^3^ -C-H), 1725 (C = O), 1691 (C = O), 1659 (C = O) and 1515 (N = N) cm-^1^^1^. H NMR (400 MHz, DMSO): δ 14.27 (s, 1 H, OH), 11.55 (s, 2H, barbituric acid 2NH), 8.23 (s, 2H, Ar-H), 7.81 (d, *J* = 8.6 Hz, 1 H, Ar-H) and 2.44 (s, 3H, CH_3_) ppm^13^. C NMR (101 MHz, DMSO): δ 150.32, 145.03, 143.97, 126.89, 126.84, 123.84, 122.98, 121.98, 115.00, 112.65, 18.20 and 16.64 (CH_3_) ppm. C_11_H_9_N_5_O_5_ requires: C, 45.36%; H, 3.09; N, 24.05%. Found: C, 45.32; H, 3.18; N, 24.15%.

**(*E*)-2-thioxo-5-(*p*-tolyldiazenyl)dihydropyrimidine-4**,**6(1*H***,**5*H*)-dione 16**.

Dark Orange crystals, 3.2 g (81.57%) yield; m.p. 320 ºC. UV: λ_max_ (DMF): 435 nm, and Ɛ_max_ 57,100 mol^-1^dm^3^cm^-1^. IR (KBr): ῡ 3433 (N-H), 3261 (N-H), 3104 (SP^2^ = C-H), 2915 (SP^3^-C-H), 1708 (C = O), 1663 (C = O), 1609 (C = S) and 1504 (N = N) cm-^1^.

**(*E*)-5-((4-methoxyphenyl)diazenyl)-2-thioxodihydropyrimidine-4**,**6(1*H***,**5*H*)-dione 17**.

Orange crystals, 3.3 g (85.20%) yield; m.p. 290 ºC. UV: λ_max_ (DMF): 440 nm, and Ɛ_max_ 32,000 mol^− 1^dm^3^cm^− 1^. IR (KBr): ῡ 3431 (N-H), 3259 (N-H), 3080 (SP^2^ = C-H), 2924 (SP^3^ -C-H), 1704 (C = O), 1659 (C = O), 1597 (C = S) and 1504 (N = N) cm-^1^.

**(*E*)-5-((4-nitrophenyl)diazenyl)-2-thioxodihydropyrimidine-4**,**6(1*H***,**5*H*)-dione 18**.

Dark brown crystals, 4.2 g (86.70%) yield; m.p. 290 ºC. UV: λmax (DMF): 415 nm, and Ɛ_max_ 51,300 mol^− 1^dm^3^cm^− 1^. IR (KBr): ῡ 3452 (N-H), 3023 (SP^2^ = C-H), 1660 (C = O) and 1529 (N = N) cm-^1^.

### General method for synthesis of dyes 19–23

A mixture of azo barbituric dye (0.5 g) and potassium carbonate (0.74 g) was dissolved in a mixture of DMSO (4 mL) and benzene (10 ml) then stirred for 15 min. at room temperature. A solution of benzyl bromide (1.8 g) in benzene (10 mL) was added slowly the mixture in about 15 min. using a dropping funnel. After the reaction was complete, the mixture was cooled to room temperature, added to 50 mL of ice-cold water, and extracted with ethyl acetate (3 × 25 mL). The combined organic layers were washed with water (2 × 20 mL), dried over anhydrous sodium sulfate, filtered, and evaporated under reduced pressure at 50 °C. The crude product was crystallized from a DMF-water mixture (1:3, 20 mL) to afford the pure dyes **19–23**.

**(*E*)-1**,**3-dibenzyl-5-(phenyldiazenyl)pyrimidine-2**,**4**,**6(1*H***,**3*H***,**5*H*)-trione 19**.

Orange crystal, 2.2 g (74.40%) yield; m.p. 200–202 ºC. UV: ג_max_ (DMF) 395 nm, and Ɛ_max_ 39,000 mol^− 1^dm^3^cm^− 1^. IR (KBr): ῡ 3034 (SP^2^ = C-H), 2966 (SP^3^ -C-H), 1725 (C = O), 1678 (C = O), 1641 (C = O) and 1525 (N = N) cm^− 1^^1^. H NMR (DMSO-*d6*, 400 MHz): δ 14.05 (s, 1 H, NH), 7.60 (d, *J* = 7.7 Hz, 2H, Ar-H), 7.43–7.37 (m, 2H, Ar-H), 7.33–7.30 (m, 4 H, Ar-H), 7.27 (t, *J* = 7.5 Hz, 4 H, Ar-H), 7.21 (t, *J* = 7.8 Hz, 3H, Ar-H) and 5.00 (s, 4 H, 2CH_2_) ppm. C_24_H_22_N_4_O_3_ requires: C, 69.55; H, 5.35; N, 13.52%. found: C, 69.59; H, 5.42; N, 13.59%.

**(*E*)-1**,**3-dibenzyl-5-(*p*-tolyldiazenyl)pyrimidine-2**,**4**,**6(1*H***,**3*H***,**5*H*)-trione 20**.

Orange crystal, 0.5 g (60.40%) yield; m.p. 210–212 ºC. UV: ג_max_ (DMF) 515 nm, and Ɛ_max_ 13,000 mol^− 1^dm^3^cm^− 1^. IR (KBr): ῡ 3031 (SP^2^ = C-H), 2956 (SP^3^ -C-H), 1701 (C = O), 1645 (C = O) and 1517 (N = N) cm^− 11^. H NMR (DMSO-*d6*, 400 MHz): δ 7.37–7.07 (m, 14 H, Ar-H), 4.93 (s, 4 H, 2CH_2_), 2.46 (s, 3H, CH_3_) ppm. C_25_H_22_N_4_O_3_ requires: C,70,41; H, 5.20; N, 13.14%. found: C, 70.48; H, 5.13; N, 13.21%.

**(*E*)-1**,**3-dibenzyl-5-((4-methoxyphenyl)diazenyl)pyrimidine-2**,**4**,**6(1*H***,**3*H***,**5*H*)-trione 21**.

Red crystal, 0.6 g (88%) yield; m.p. 190–192 ºC. UV: ג_max_ (DMF) 415 nm, and Ɛ_max_ 42,000 mol^− 1^dm^3^cm^− 1^. IR (KBr): ῡ 3060 (SP^2^ = C-H), 2931 (SP^3^ -C-H), 1654 (C = O) and 1528 (N = N) cm^− 11^. H NMR (DMSO-*d6*, 400 MHz): δ 7.62 (d, 2H, *J* = 7.7 Hz, Ar-H), 7.34 (m, 8 H, Ar-H), 7.26 (t, *J* = 7.5 Hz, 2H, Ar-H), 7.04 (d, *J* = 7.8 Hz, 2H, Ar-H), 5.05 (s, 4 H, 2CH_2_) and 3.80 (s, 3H, OCH_3_) ppm^13^. C NMR (APT) (DMSO-*d6*, 101 MHz): δ 158.55 (C = O), 151.17 (C = O), 137.71, 129.05, 128.77, 127.83, 127.55, 127.49, 119.56, 116.17, 115.25, 55.91 (NCH_2_) and 44.33 (OCH_3_) ppm. C_25_H_22_N_4_O_4_ requires: C, 67.86; H, 5.01; N, 12.66%. found: C, 67.92; H, 5.14; N, 12.52%.

**(*E*)-1**,**3-dibenzyl-5-((4-chlorophenyl)diazenyl)pyrimidine-2**,**4**,**6(1*H***,**3*H***,**5*H*)-trione 22**.

Red crystal, 1.0 g (65.40%) yield; m.p. 220–222 ºC. UV: ג_max_ (DMF) 385 nm, and Ɛ_max_ 90,000 mol^− 1^dm^3^cm^− 1^. IR (KBr): ῡ 3031 (SP^2^ = C-H), 2961 (SP^3^ -C-H), 1686 (C = O), 1630 (C = O) and 1518 (N = N) cm^− 1^^1^. H NMR (DMSO-*d6*, 400 MHz): δ 7.60 (m, 2H, Ar-H), 7.54 (m, 3H, Ar-H), 7.26 (d, *J* = 7.6 Hz, 2H, Ar-H), 7.31 (m, 5 H, Ar-H), 7.23 (m, 3H, Ar-H) and 4.59 (s, 4 H, 2CH_2_) ppm^13^. C NMR (APT) (DMSO-*d6*, 101 MHz): δ 139.04, 133.63, 130.84, 129.96, 129.46, 129.16, 128.62, 128.39, 127.75, 127.48, 127.18 and 48.64 (CH_2_) ppm. C_24_H_21_ClN_4_O_3_ requires: C, 64.21; H, 4.72; N, 12.48%. found: C, 64.27; H, 4.78; N, 12.52%.

**(*E*)-1**,**3-dibenzyl-5-((4-nitrophenyl)diazenyl)pyrimidine-2**,**4**,**6(1*H***,**3*H***,**5*H*)-trione 23**.

Brown crystal, 0.7 g (62.0%) yield; m.p. 170–172 ºC. UV: ג_max_ (DMF) 405 nm, and Ɛ_max_ 48,000 mol^− 1^dm^3^cm^− 1^. IR (KBr): ῡ 3031 (SP^2^ = C-H), 2956 (SP^3^ -C-H), 1727 (C = O), 1681 (C = O) and 1515 (N = N) cm^− 1^^1^. H NMR (DMSO-*d6*, 400 MHz): δ 14.00 (s, H, NH), 8.32 (m, 2H, Ar-H), 8.00 (m, 2H, Ar-H), 7.88 (m, 3H, Ar-H), 7.40 (m, 3H, Ar-H), 7.33 (m, 2H, Ar-H), 7.26 (m, 2H, Ar-H) and 5.08 (s, 4 H, 2CH_2_) ppm^13^. C NMR (APT) (DMSO-*d6*, 101 MHz): δ 156.19, 150.91, 144.59, 128.78, 128.65, 127.94, 127.69, 126.89, 126.04, 117.65, 112.85 and 46.30 (CH_2_) ppm. C_24_H_21_N_5_O_5_ requires: C, 62.74; H, 4.61; N, 15.24%. found: C, 62.64; H, 4.53; N, 15.32%.

## Electronic supplementary material

Below is the link to the electronic supplementary material.

Supplementary Material 1
