# Supplementary material for: Synthesis, characterization, and polyester dyeing performance of azo barbituric and thiobarbituric acid disperse dyes
Source: Sci Rep. 2025 Apr 18;15:13410. doi: 10.1038/s41598-025-96473-x (PMC12008282; doi:10.1038/s41598-025-96473-x)
Supplement: Supplementary file 1 — Supplementary Material 1 [file 41598_2025_96473_MOESM1_ESM.docx]

**Synthesis, Characterization, and Polyester Dyeing Performance of Azo Barbituric and Thiobarbituric Acid Disperse Dyes**

Mohamed A. El-Rahman^a^, Alaa Z. Omar^a,*^, Alshimaa R. Kandeel^b^_,_ Ezzat A. Hamed^a^, Mohamed A. El-Atawy^a^ and Reda M. Keshk^b^

^a^ Chemistry Department, Faculty of Science, Alexandria University, P.O. 426 Ibrahemia, Alexandria 21321, Egypt

^b^ Chemistry Department, Faculty of Science, Damanhour University, Damanhour, Egypt

*Corresponding author: [Alaazaki@alexu.edu.eg](mailto:Alaazaki@alexu.edu.eg), Tel.: +201111361784

Materials and Instruments:

All reagents and solvents used in the synthesis were of analytical grade, purchased from Merck (Germany), and used exactly as received. Melting points were determined by MEL-TEMP II melting point apparatus in open glass capillaries. The IR spectra were recorded as potassium bromide (KBr) discs on a Perkin-Elemer FT-IR (Fourier-Transform Infrared Spectroscopy), Faculty of Science, Alexandria University. The NMR spectra were carried out at ambient temperature (~25 ºC) on a (JEOL) 500 MHz spectrophotometer using tetramethylsilane (TMS) as an internal standard, NMR Unit, Faculty of Science, Mansoura University. Elemental analyses were analyzed at the Regional Center for Mycology and Biotechnology, Al-Azhar University, Cairo, Egypt. All dyeing processes were carried out using a laboratory sample dyer (DL-6000P/S-3) (starlet-3).

Equations used in the calculation of electronic parameters:

| IP = − E_HOMO_ |
| --- |
| EA = − E_LUMO_ |
| ΔE_LUMO-HOMO_ = − E_LUMO_–(− E _HOMO_) = E _HOMO_ - E_LUMO_ |
| χ = IP + EA / 2 |
| μ = − χ |
| η = IP – EA/ 2 |
| σ = 1/η |
| ω = μ^2^/2η |


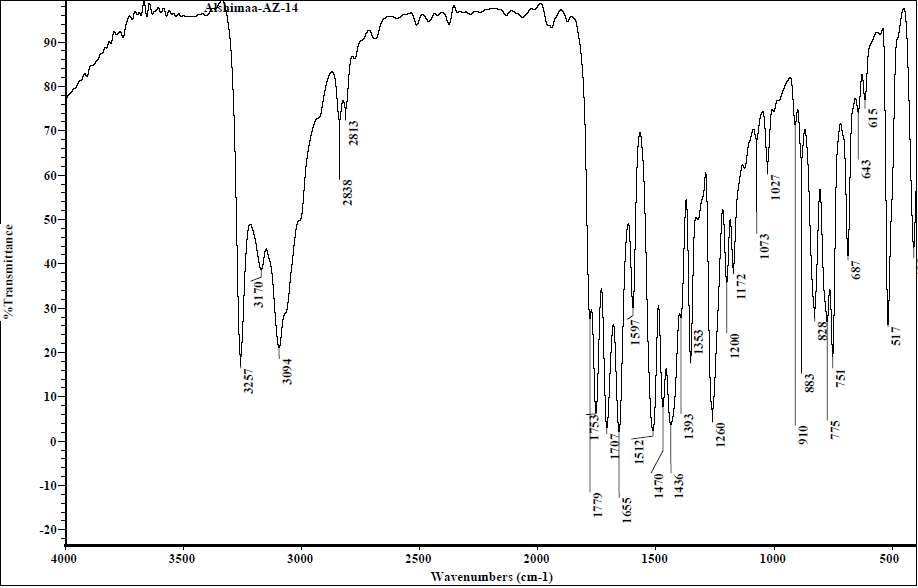


**Figure S1. IR (KBr) spectrum of (*E*)-5-(phenyldiazenyl)pyrimidine-2,4,6(1*H*,3*H*,5*H*)-trione** **1**


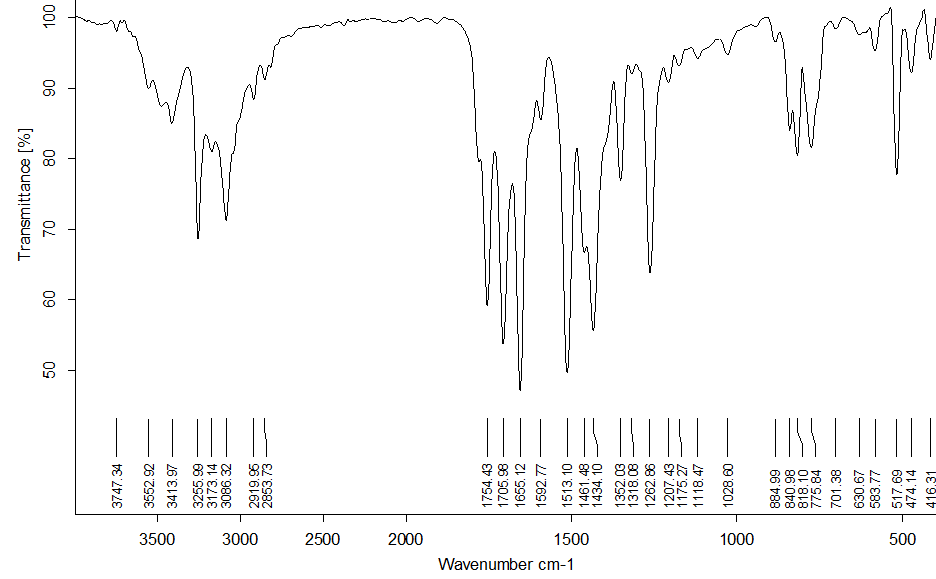


**Figure S2. IR (KBr) spectrum of (*E*)-5-(*p*-tolyldiazenyl)pyrimidine-2,4,6(1*H*,3*H*,5*H*)-trione 2**


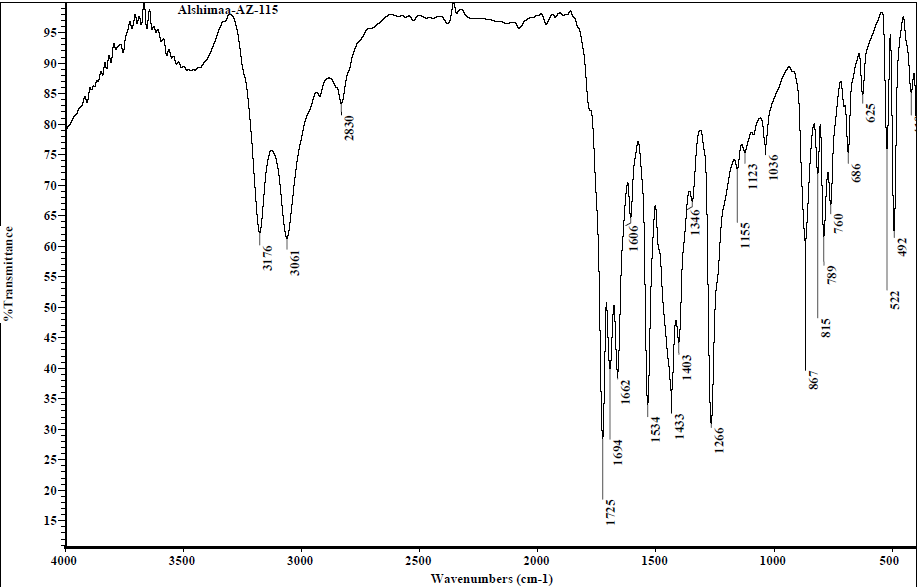


**Figure S3. IR (KBr) spectrum of (*E*)-5-(*m*-tolyldiazenyl)pyrimidine-2,4,6(1*H*,3*H*,5*H*)-trione 3**

 **Figure S4. ^1^H NMR (400 MHz, DMSO-*d6*) spectrum of (*E*)-5-(*m*-tolyldiazenyl)pyrimidine-2,4,6(1*H*,3*H*,5*H*)-trione 3**


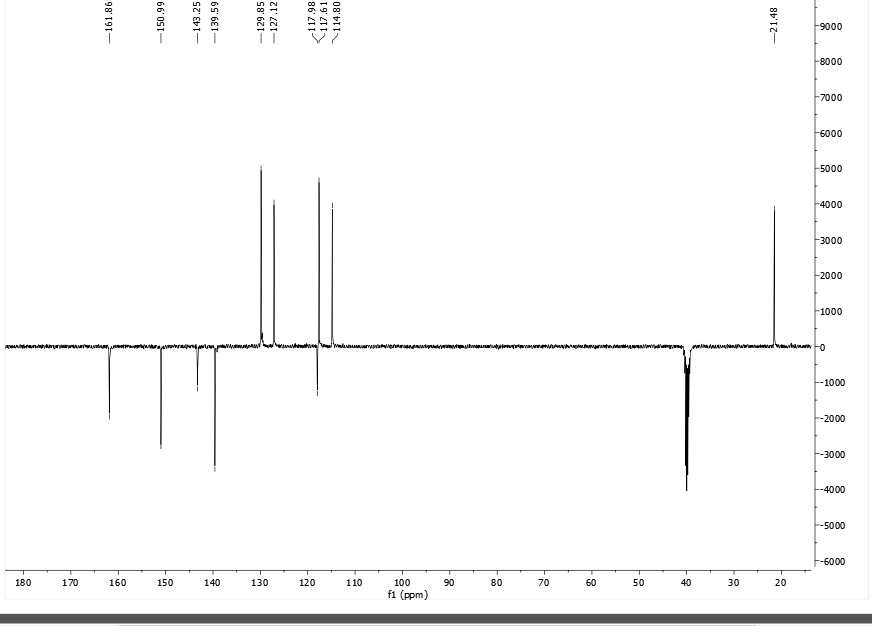


**Figure S5. ^13^C NMR (400 MHz, DMSO-*d6*) spectrum of (*E*)-5-(*m*-tolyldiazenyl)pyrimidine-2,4,6(1*H*,3*H*,5*H*)-trione 3**


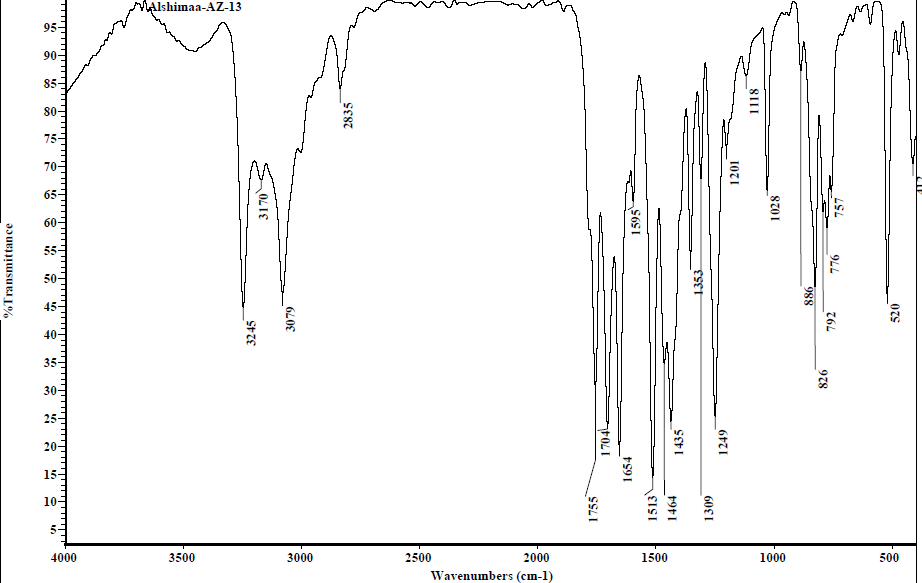


**Figure S6. IR (KBr) spectrum of (*E*)-5-((4-methoxyphenyl)diazenyl)pyrimidine-2,4,6(1*H*,3*H*,5*H*)-trione 4**


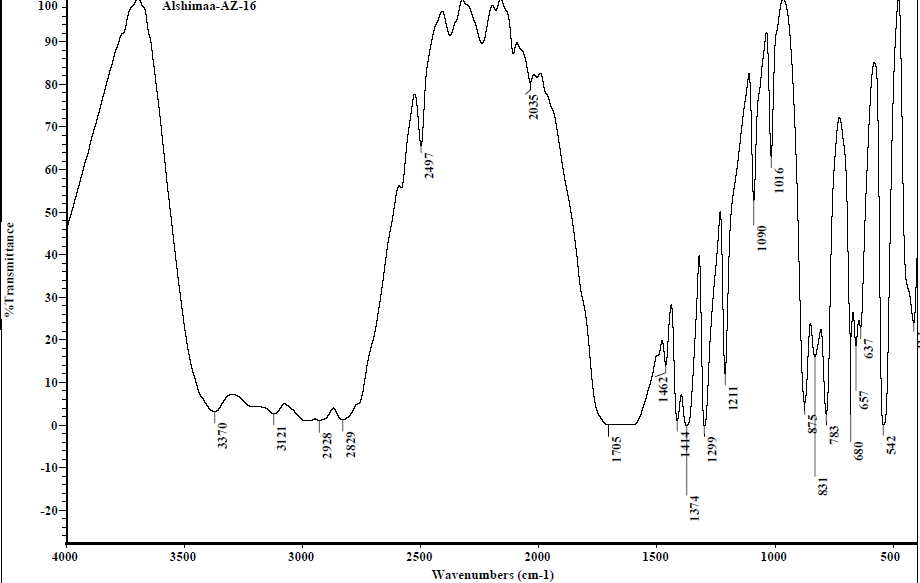


**Figure S7. IR (KBr) spectrum of (*E*)-5-((4-hydroxyphenyl)diazenyl)pyrimidine-2,4,6(1*H*,3*H*,5*H*)-trione 5**


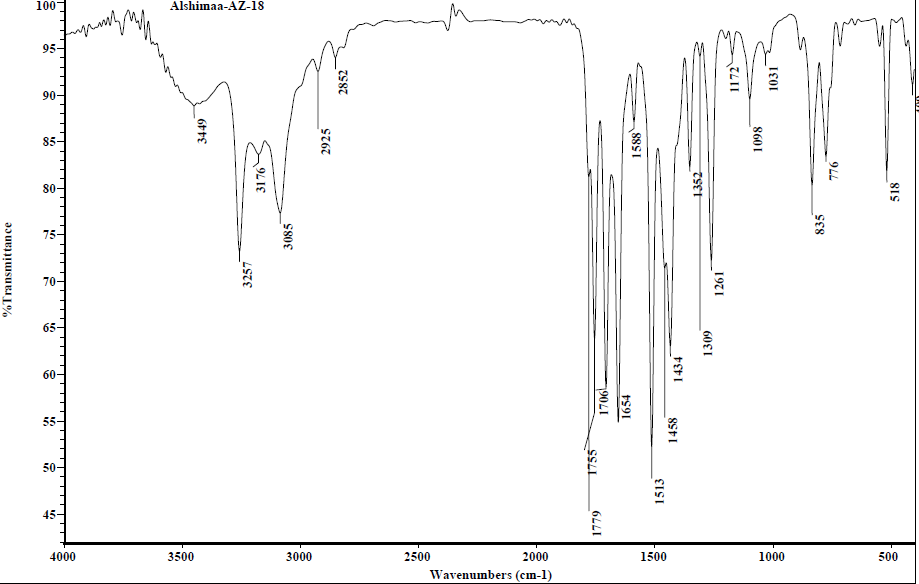


**Figure S8. IR (KBr) spectrum of (*E*)-5-((4-chlorophenyl)diazenyl)pyrimidine-2,4,6(1*H*,3*H*,5*H*)-trione 6**


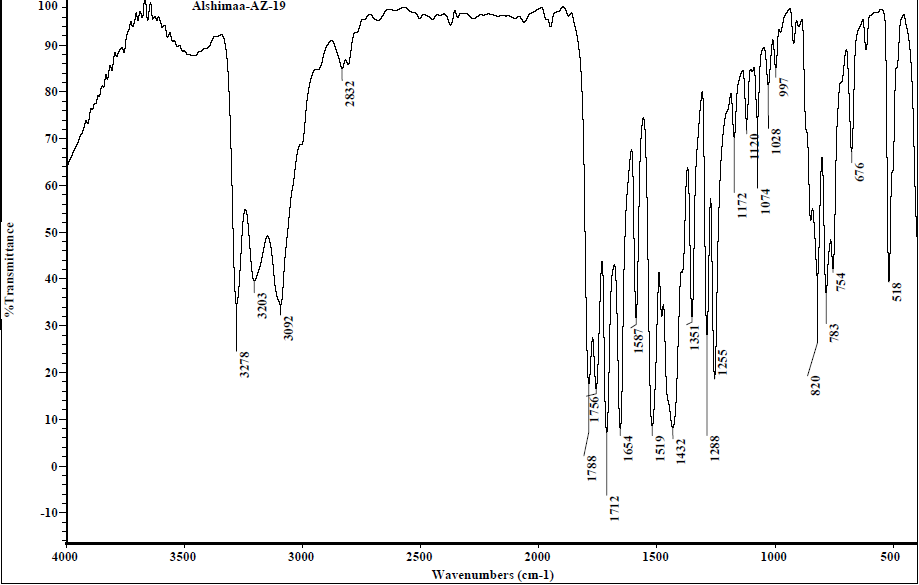


**Figure S9. IR (KBr) spectrum of (*E*)-5-((3-chlorophenyl)diazenyl)pyrimidine-2,4,6(1*H*,3*H*,5*H*)-trione 7**

**Figure S10. ^1^H NMR (400 MHz, DMSO-*d6*) spectrum of (*E*)-5-((3-chlorophenyl)diazenyl)pyrimidine-2,4,6(1*H*,3*H*,5*H*)-trione 7**

**Figure S11. ^1^H NMR (400 MHz, DMSO-*d6*) spectrum of (*E*)-5-((3-chlorophenyl)diazenyl)pyrimidine-2,4,6(1*H*,3*H*,5*H*)-trione 7**


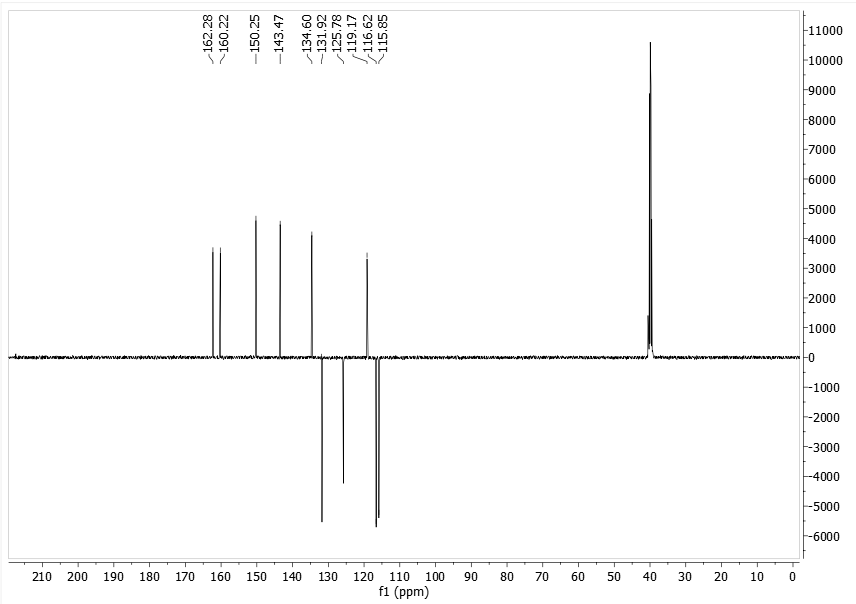


**Figure S12. ^13^C NMR (101 MHz, DMSO-*d6*) spectrum of (*E*)-5-((3-chlorophenyl)diazenyl)pyrimidine-2,4,6(1*H*,3*H*,5*H*)-trione 7**


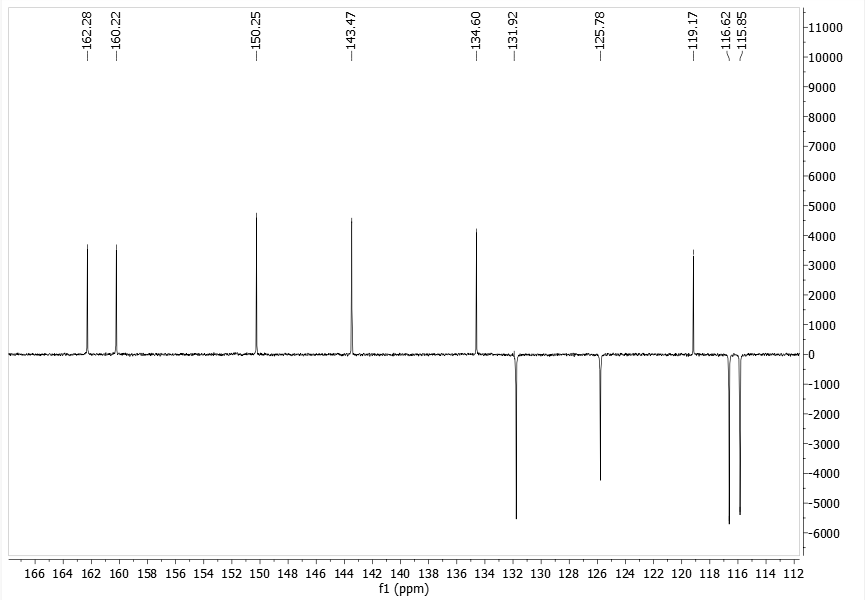


**Figure S13. ^13^C NMR (101 MHz, DMSO-*d6*) spectrum of (*E*)-5-((3-chlorophenyl)diazenyl)pyrimidine-2,4,6(1*H*,3*H*,5*H*)-trione 7**


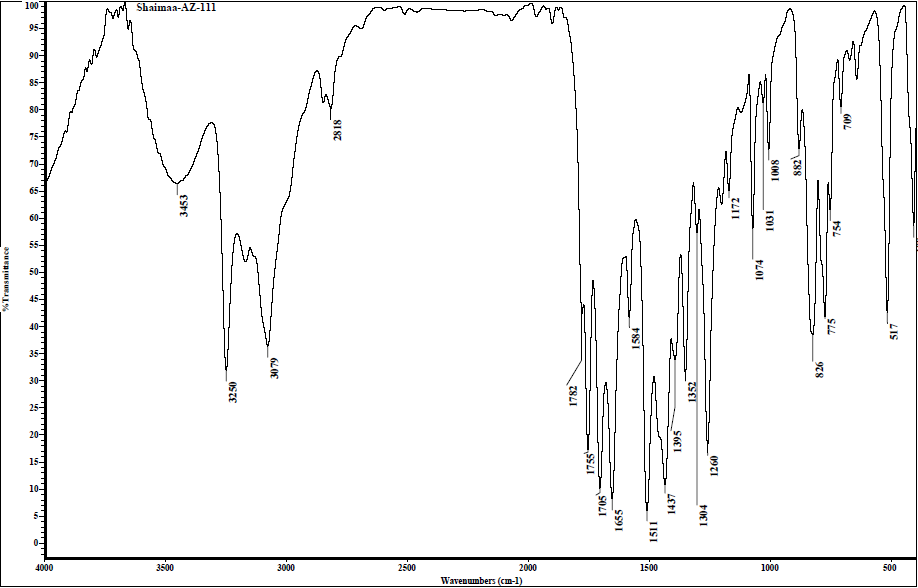


**Figure S14. IR (KBr) spectrum of (*E*)-5-((4-bromophenyl)diazenyl)pyrimidine-2,4,6(1*H*,3*H*,5*H*)-trione 8**


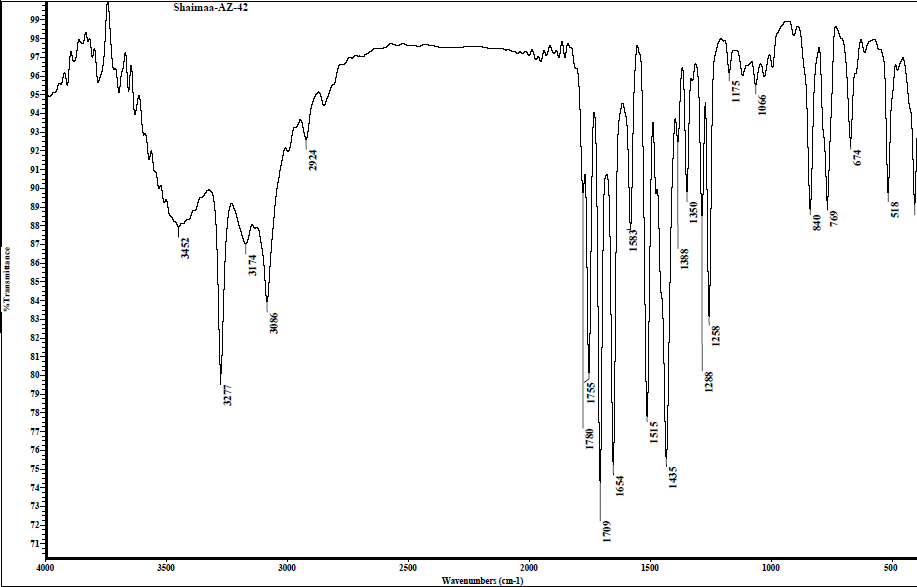


**Figure S15. IR (KBr) spectrum of (*E*)-5-((3-bromophenyl)diazenyl)pyrimidine-2,4,6(1*H*,3*H*,5*H*)-trione 9**


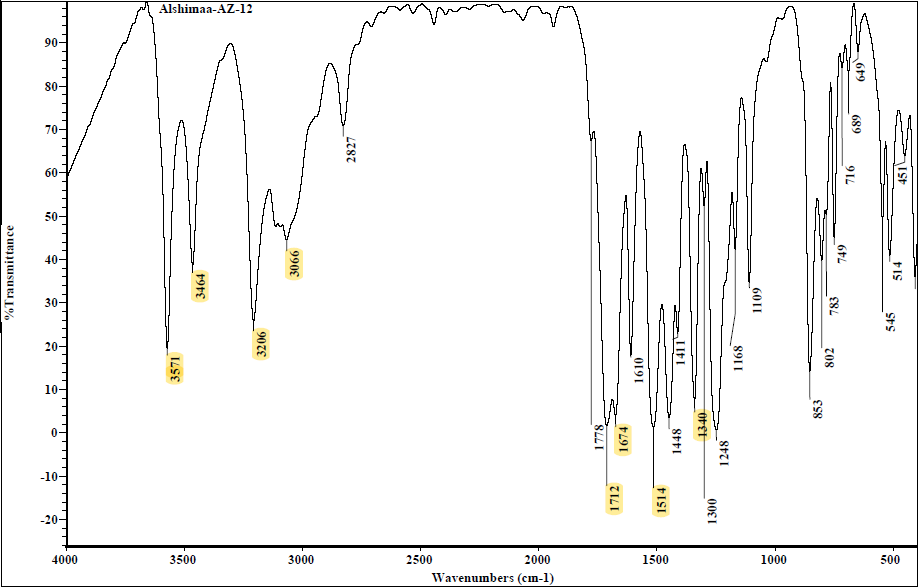


**Figure S16. IR (KBr) spectrum of (*E*)-5-((4-nitrophenyl)diazenyl)pyrimidine-2,4,6(1*H*,3*H*,5*H*)-trione 10**


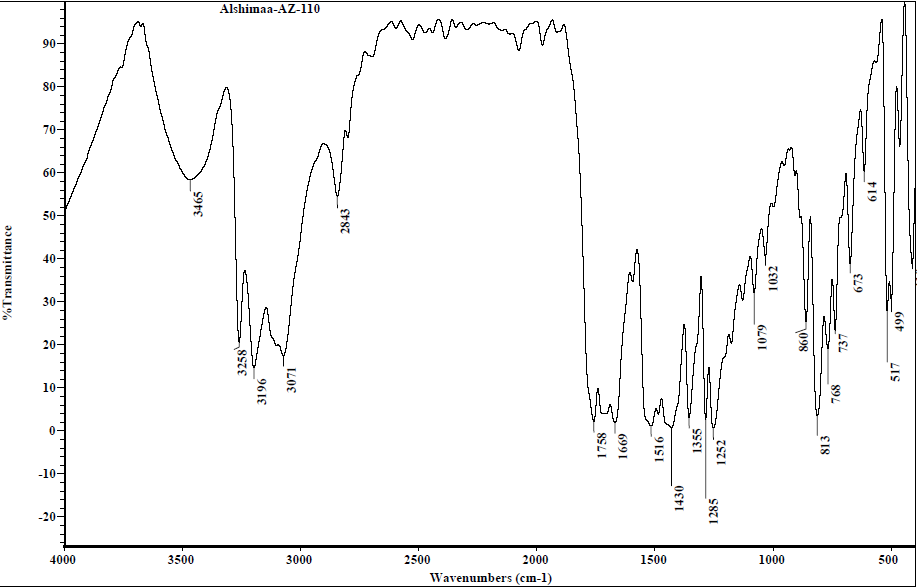


**Figure S17. IR (KBr) spectrum of (*E*)-5-((3-nitrophenyl)diazenyl)pyrimidine-2,4,6(1*H*,3*H*,5*H*)-trione 11**


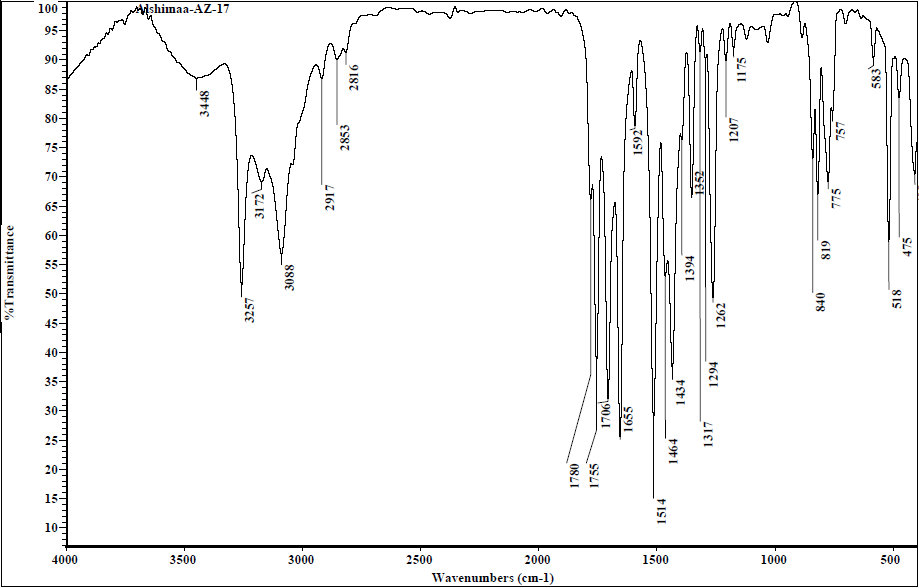


**Figure S18. IR (KBr) spectrum of (*E*)-4-((2,4,6-trioxohexahydropyrimidin-5-yl)diazenyl)benzenesulfonic acid 12**


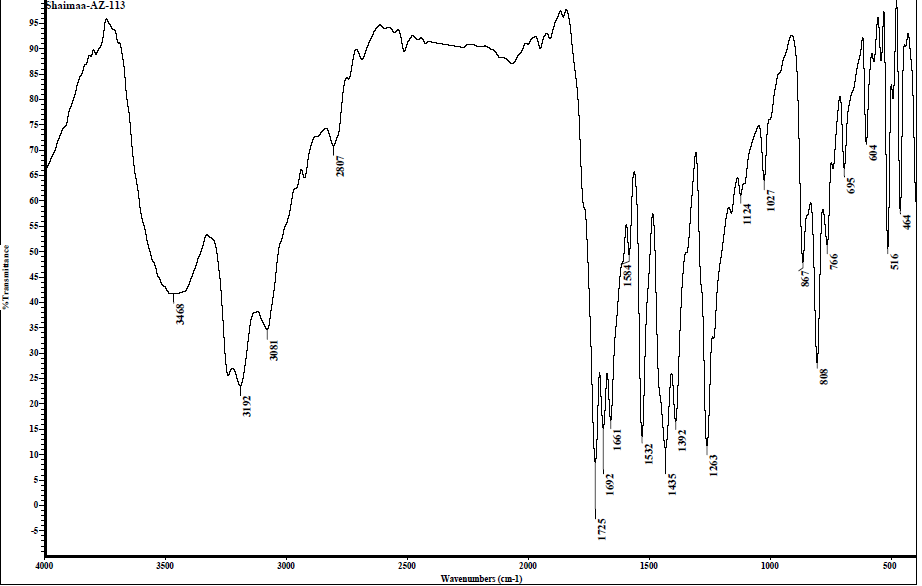


**Figure S19. IR (KBr) spectrum of (*E*)-5-((3,4-dimethylphenyl)diazenyl)pyrimidine-2,4,6(1*H*,3*H*,5*H*)-trione 13**

**Figure S20. ^1^H NMR (400 MHz, DMSO-*d6*) spectrum of (*E*)-5-((3,4-dimethylphenyl)diazenyl)pyrimidine-2,4,6(1*H*,3*H*,5*H*)-trione 13**

**Figure S21. ^1^H NMR (400 MHz, DMSO-*d6*) spectrum of (*E*)-5-((3,4-dimethylphenyl)diazenyl)pyrimidine-2,4,6(1*H*,3*H*,5*H*)-trione 13**

**Figure S22. ^1^H NMR (400 MHz, DMSO-*d6*) spectrum of (*E*)-5-((3,4-dimethylphenyl)diazenyl)pyrimidine-2,4,6(1*H*,3*H*,5*H*)-trione 13**


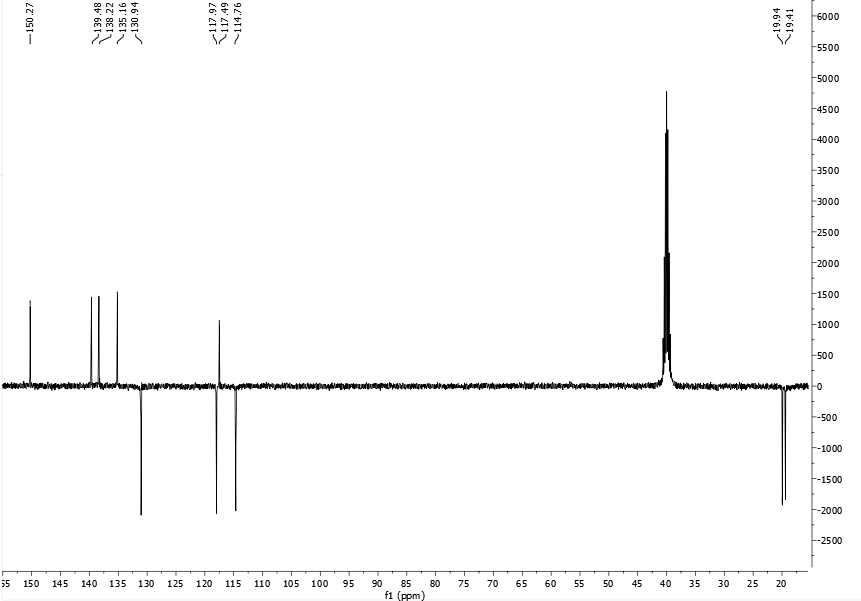


**Figure S23. ^13^C NMR (101 MHz, DMSO-*d6*) spectrum of (*E*)-5-((3,4-dimethylphenyl)diazenyl)pyrimidine-2,4,6(1*H*,3*H*,5*H*)-trione 13**


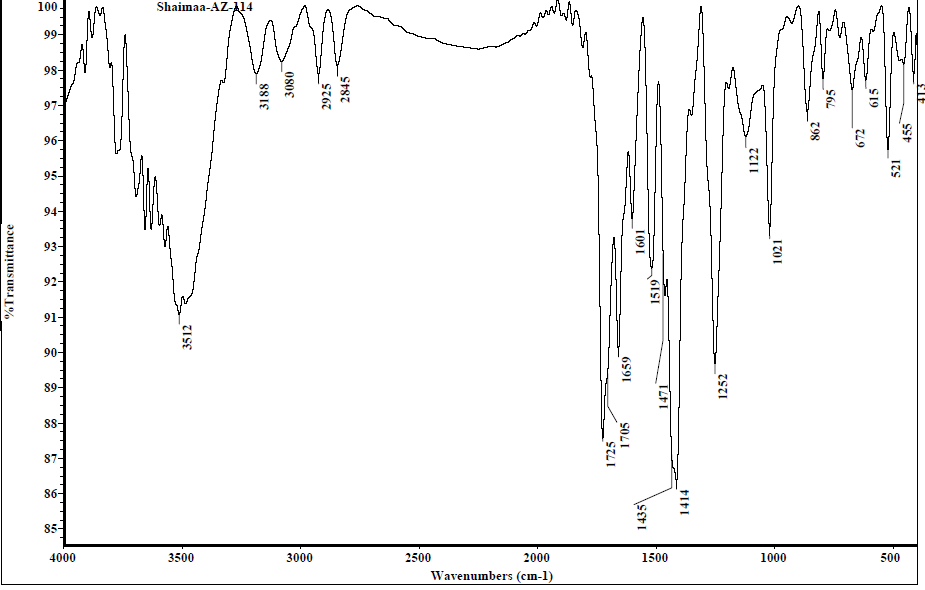


**Figure S24. IR (KBr) spectrum of (*E*)-5-((3,4-dimethoxyphenyl)diazenyl)pyrimidine-2,4,6(1*H*,3*H*,5*H*)-trione 14**

**Figure S25. ^1^H NMR (400 MHz, DMSO-*d6*) spectrum of (*E*)-5-((3,4-dimethoxyphenyl)diazenyl)pyrimidine-2,4,6(1*H*,3*H*,5*H*)-trione 14**

**Figure S26. ^1^H NMR (400 MHz, DMSO-*d6*) spectrum of (*E*)-5-((3,4-dimethoxyphenyl)diazenyl)pyrimidine-2,4,6(1*H*,3*H*,5*H*)-trione 14**


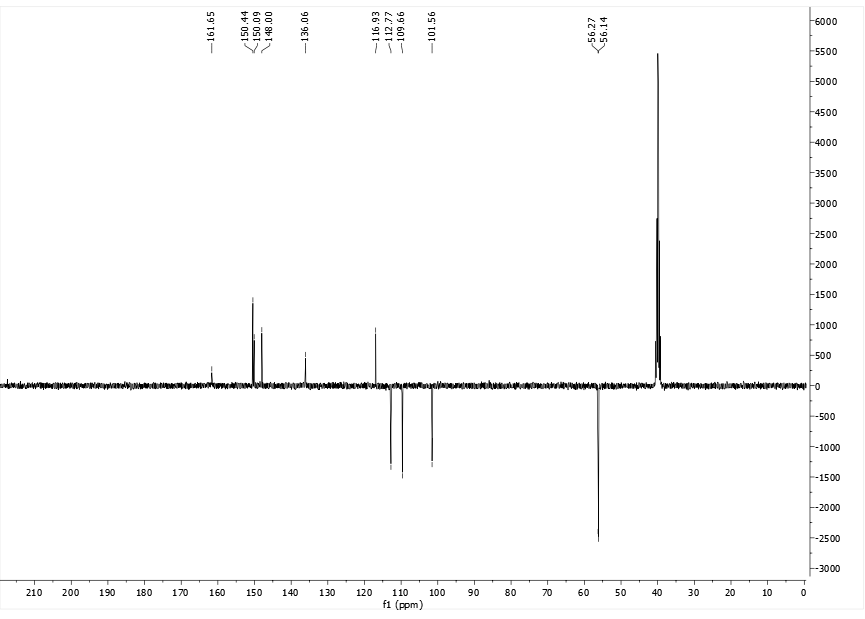


**Figure S27. ^13^C NMR (101 MHz, DMSO-*d6*) spectrum of (*E*)-5-((3,4-dimethoxyphenyl)diazenyl)pyrimidine-2,4,6(1*H*,3*H*,5*H*)-trione 14**


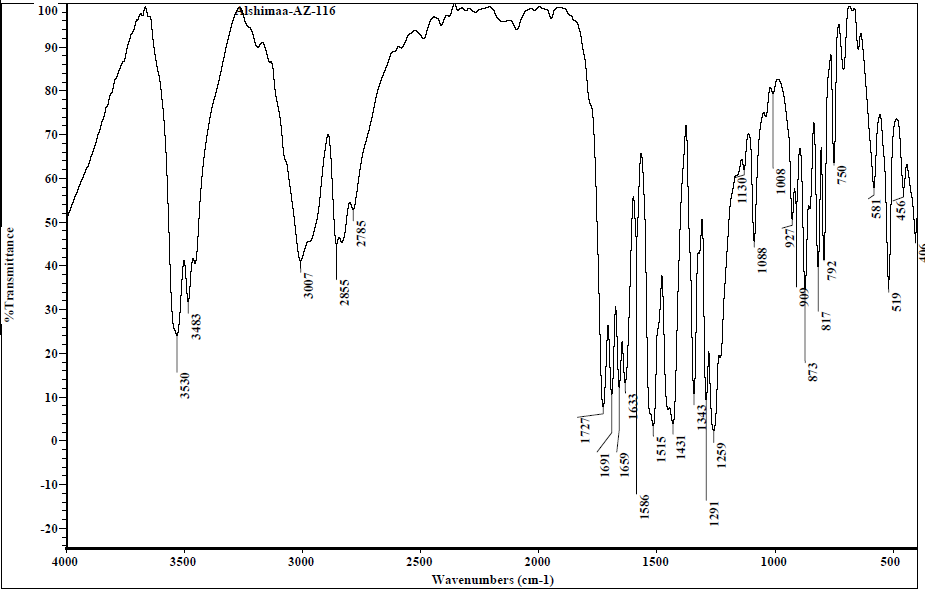


**Figure S28. IR (KBr) spectrum of (*E*)-5-((2-methyl-4-nitrophenyl)diazenyl)pyrimidine-2,4,6(1*H*,3*H*,5*H*)-trione 15**

**Figure S29. ^1^H NMR (400 MHz, DMSO-*d6*) spectrum of (*E*)-5-((2-methyl-4-nitrophenyl)diazenyl)pyrimidine-2,4,6(1*H*,3*H*,5*H*)-trione 15**


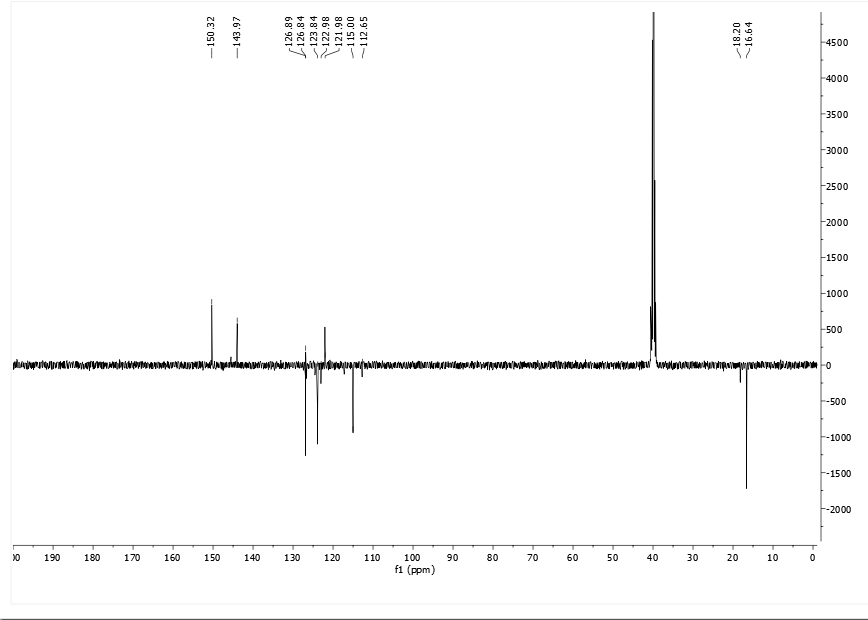


**Figure S30. ^13^C NMR (101 MHz, DMSO-*d6*) spectrum of (*E*)-5-((2-methyl-4-nitrophenyl)diazenyl)pyrimidine-2,4,6(1*H*,3*H*,5*H*)-trione 15**


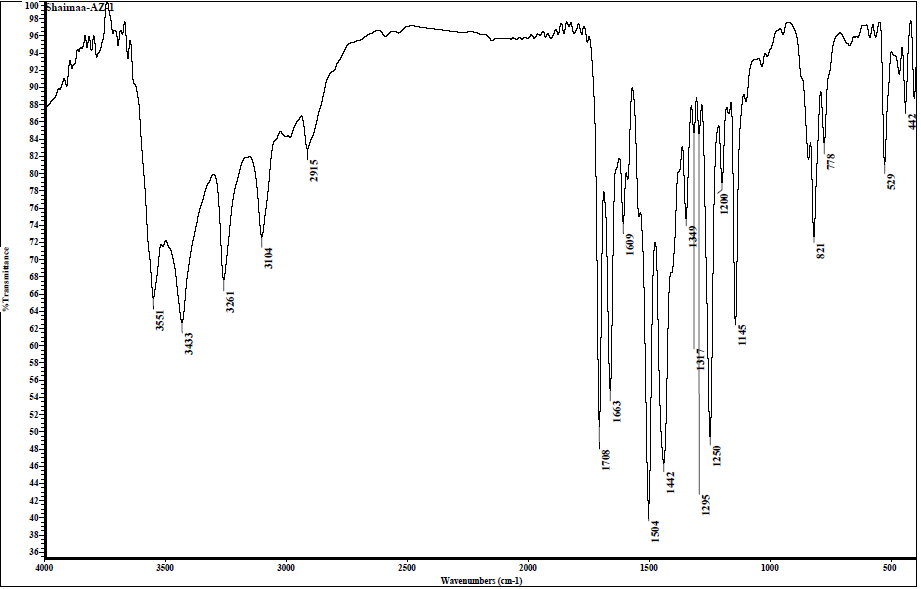


**Figure S31. IR (KBr) spectrum of (*E*)-2-thioxo-5-(*p*-tolyldiazenyl)dihydropyrimidine-4,6(1*H*,5*H*)-dione 16**


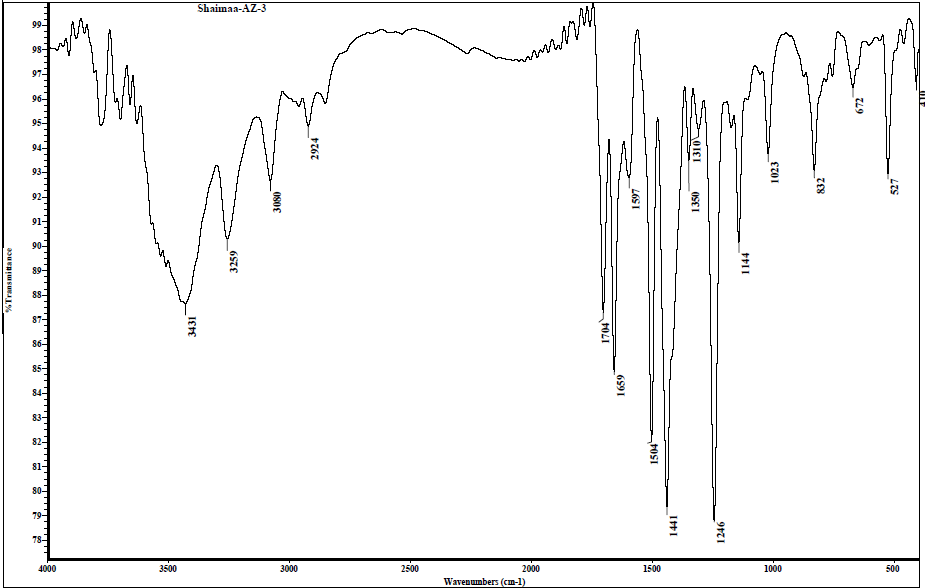


**Figure S32. IR (KBr) spectrum of (*E*)-5-((4-methoxyphenyl)diazenyl)-2-thioxodihydropyrimidine-4,6(1*H*,5*H*)-dione 17**


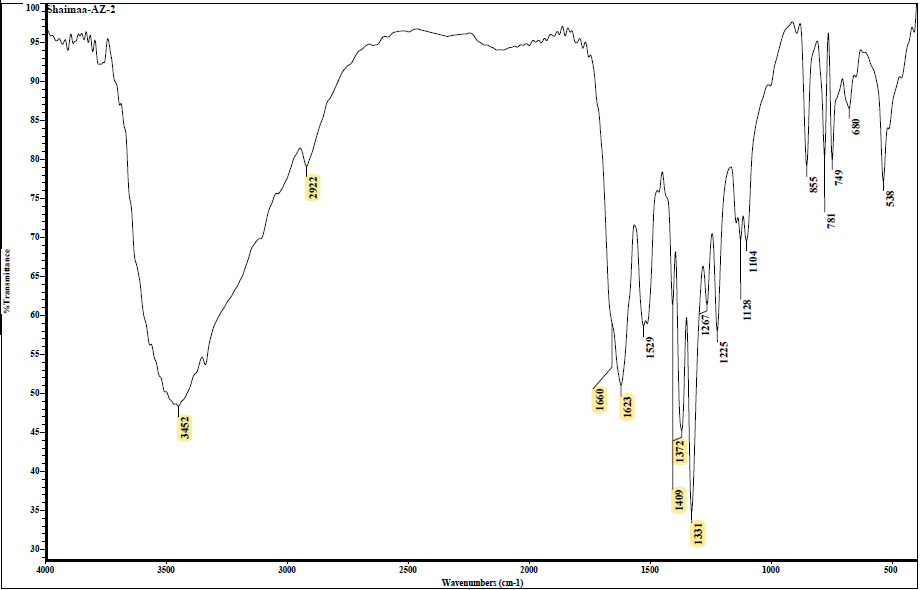


**Figure S33. IR (KBr) spectrum of (*E*)-5-((4-nitrophenyl)diazenyl)-2-thioxodihydropyrimidine-4,6(1*H*,5*H*)-dione 18**

^
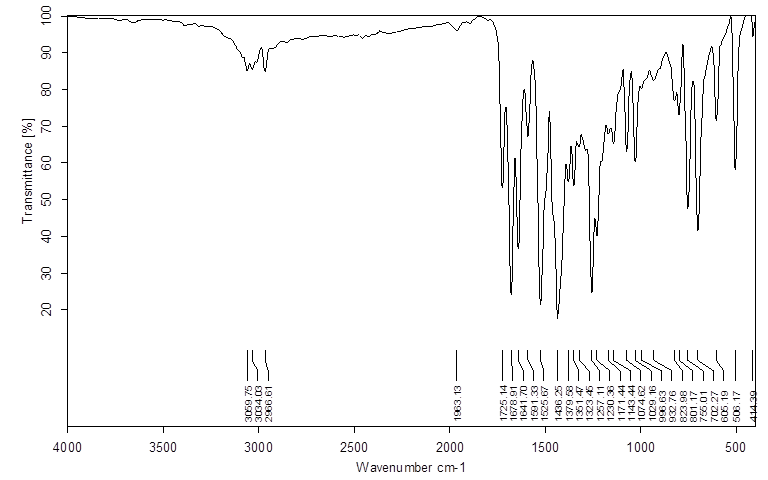
^

**Figure S34. IR (KBr) spectrum of (*E*)-1,3-dibenzyl-5-(phenyldiazenyl)pyrimidine-2,4,6(1*H*,3*H*,5*H*)-trione 19**

^
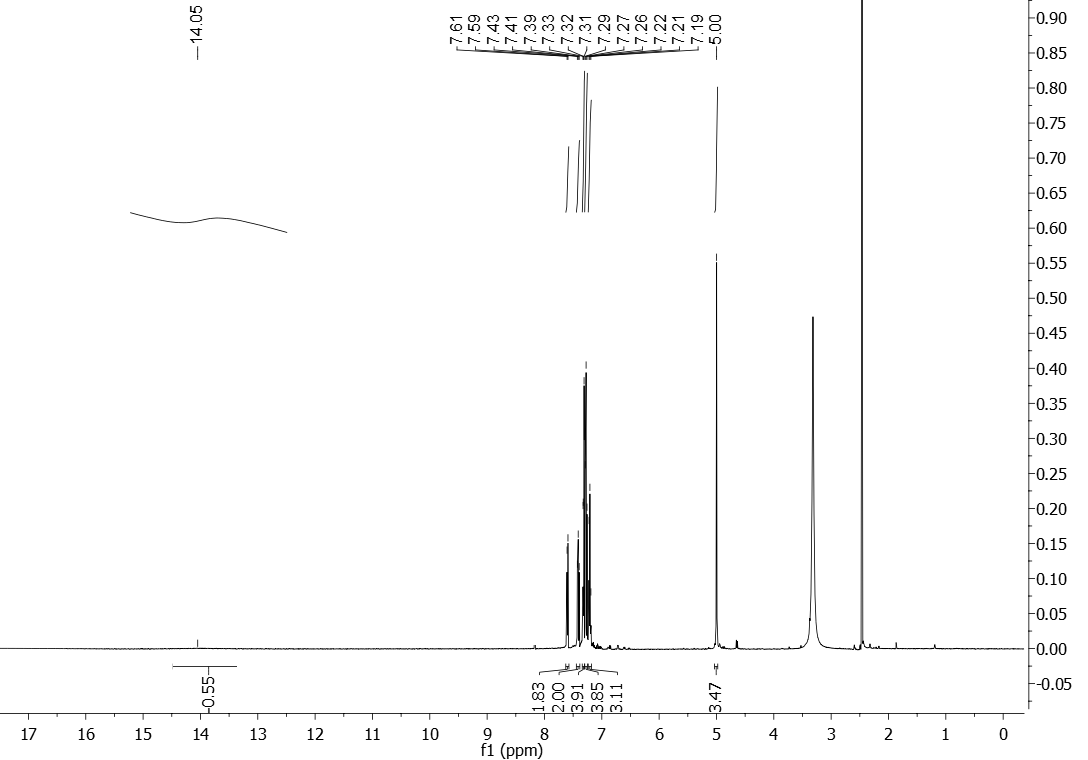
^

**Figure S35. ^1^H NMR (DMSO-*d6*) spectrum of (*E*)-1,3-dibenzyl-5-(phenyldiazenyl)pyrimidine-2,4,6(1*H*,3*H*,5*H*)-trione 19**


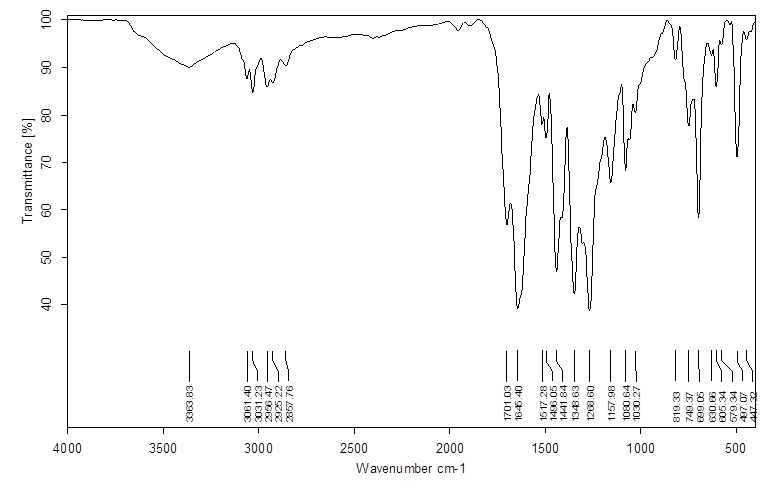


**Figure S36. IR (KBr) spectrum of (*E*)-1,3-dibenzyl-5-(*p*-tolyldiazenyl)pyrimidine-2,4,6(1*H*,3*H*,5*H*)-trione 20**

**^
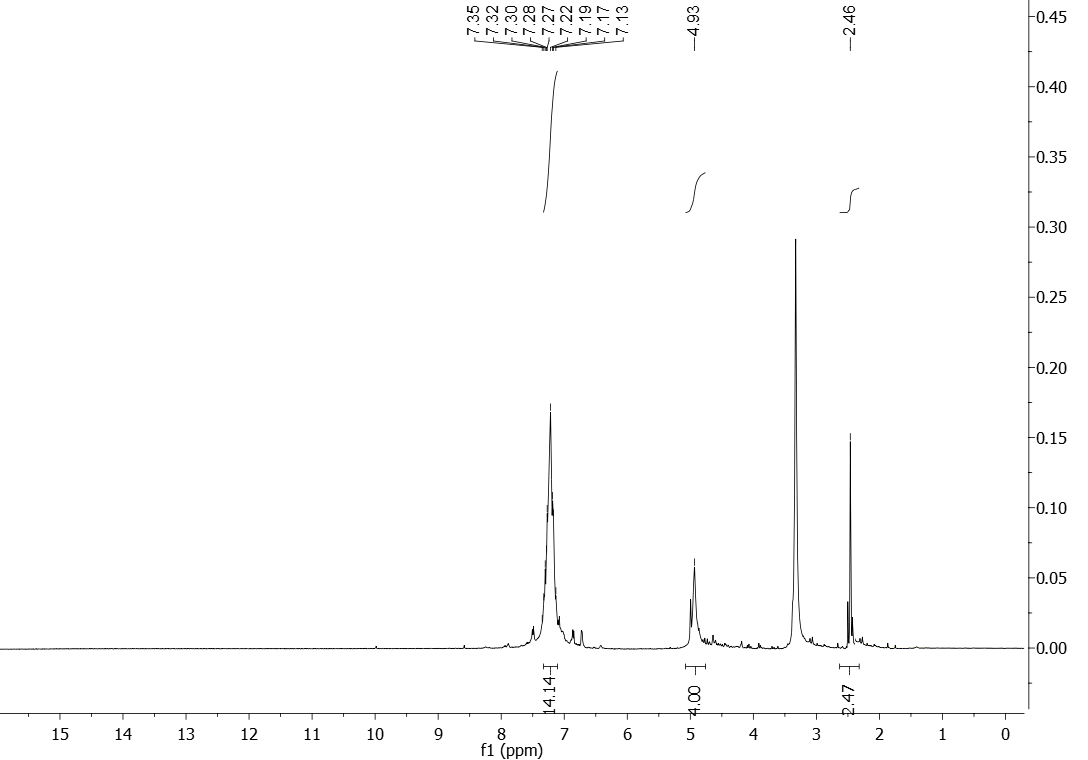
^**

**Figure S37. ^1^H NMR (DMSO-*d6*) spectrum of (*E*)-1,3-dibenzyl-5-(*p*-tolyldiazenyl)pyrimidine-2,4,6(1*H*,3*H*,5*H*)-trione 20**


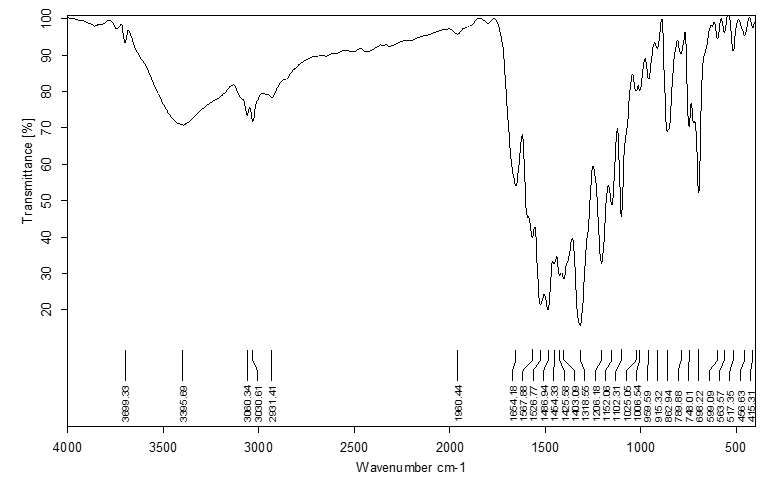


**Figure S38. IR (KBr) spectrum of (*E*)-1,3-dibenzyl-5-((4-methoxyphenyl)diazenyl)pyrimidine-2,4,6(1*H*,3*H*,5*H*)-trione 21**

**^
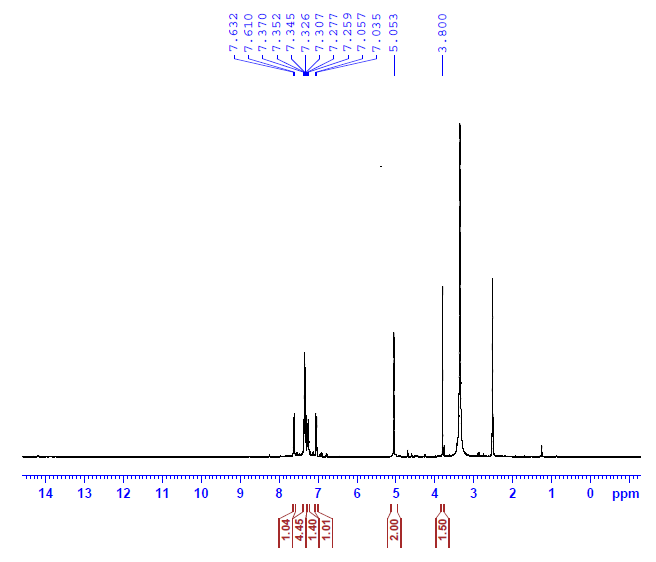
^**

**Figure S39. ^1^H NMR (DMSO-*d6*) spectrum of (*E*)-1,3-dibenzyl-5-((4-methoxyphenyl)diazenyl)pyrimidine-2,4,6(1*H*,3*H*,5*H*)-trione 21**

^^

**Figure S40. ^13^C NMR (DMSO-*d6*) of (*E*)-1,3-dibenzyl-5-((4-methoxyphenyl)diazenyl)pyrimidine-2,4,6(1*H*,3*H*,5*H*)-trione 21**

**
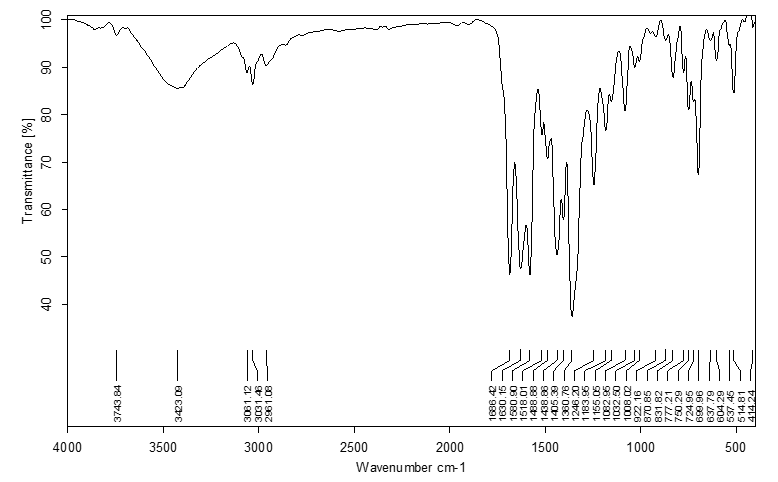
**

**Figure S41. IR (KBr) spectrum of (*E*)-1,3-dibenzyl-5-((4-chlorophenyl)diazenyl)pyrimidine-2,4,6(1*H*,3*H*,5*H*)-trione 22**

**^
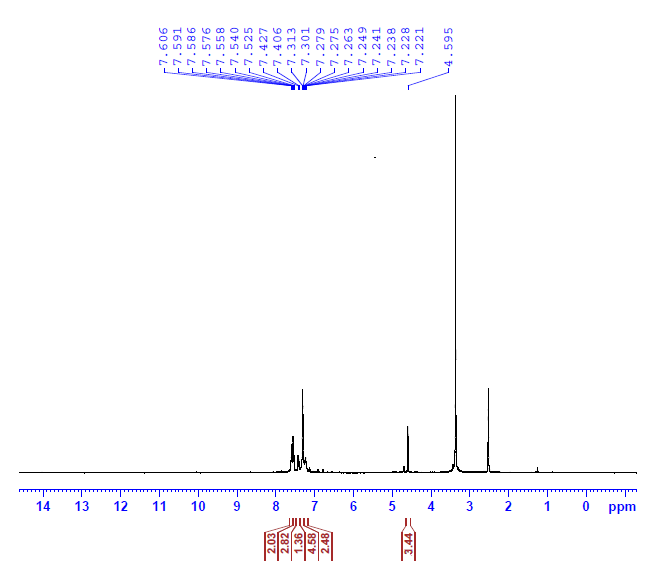
^**

**Figure S42. ^1^H NMR (DMSO-*d6*) spectrum of (*E*)-1,3-dibenzyl-5-((4-chlorophenyl)diazenyl)pyrimidine-2,4,6(1*H*,3*H*,5*H*)-trione 22**

^
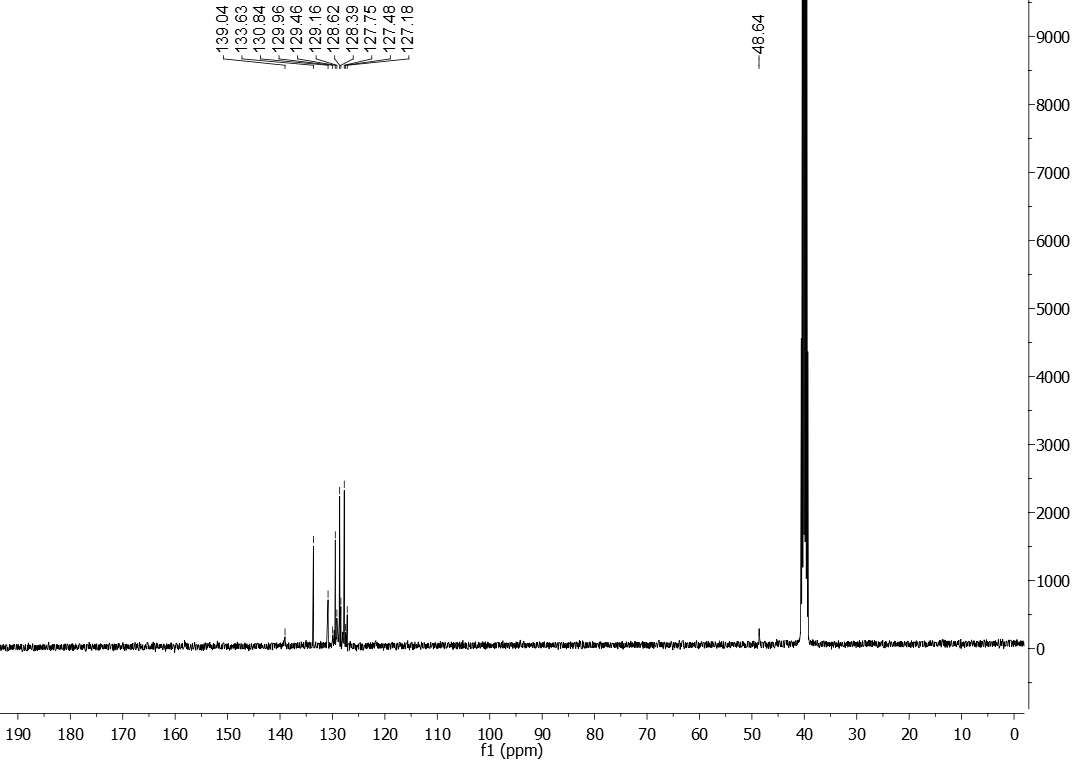
^

**Figure S43. ^13^C NMR (DMSO-*d6*) of (*E*)-1,3-dibenzyl-5-((4-chlorophenyl)diazenyl)pyrimidine-2,4,6(1*H*,3*H*,5*H*)-trione 22**

**
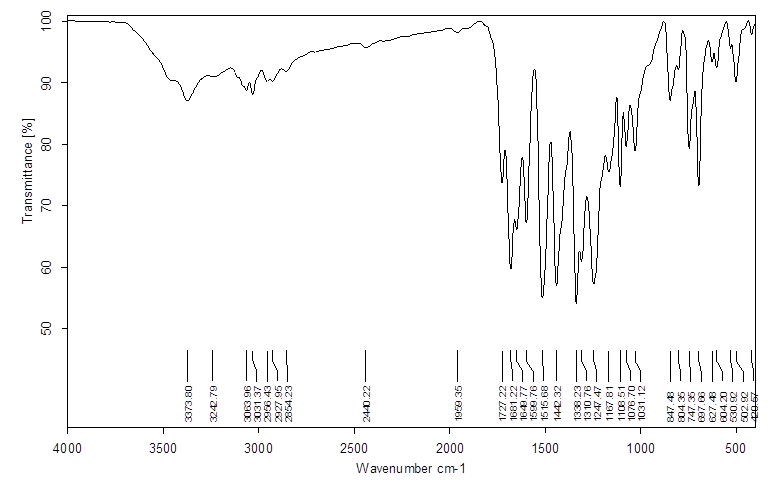
**

**Figure S44. IR (KBr) spectrum of (*E*)-1,3-dibenzyl-5-((4-nitrophenyl)diazenyl)pyrimidine-2,4,6(1*H*,3*H*,5*H*)-trione 23**

**^
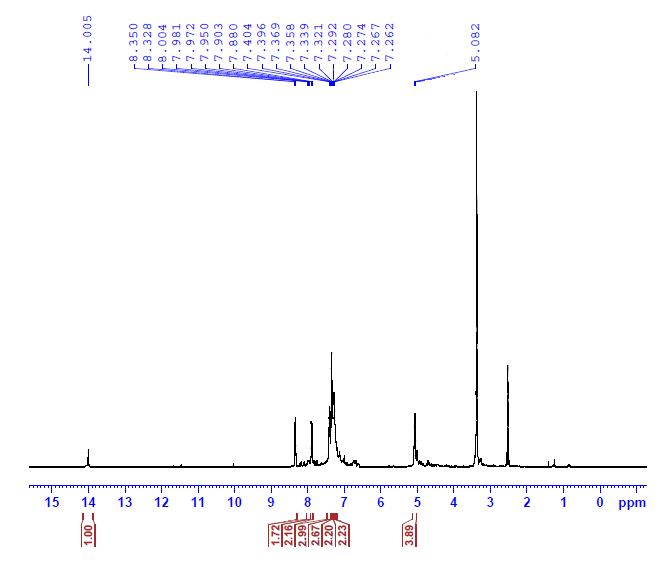
^**

**Figure S45. ^1^H NMR (DMSO-*d6*) spectrum of (*E*)-1,3-dibenzyl-5-((4-nitrophenyl)diazenyl)pyrimidine-2,4,6(1*H*,3*H*,5*H*)-trione 23**

^
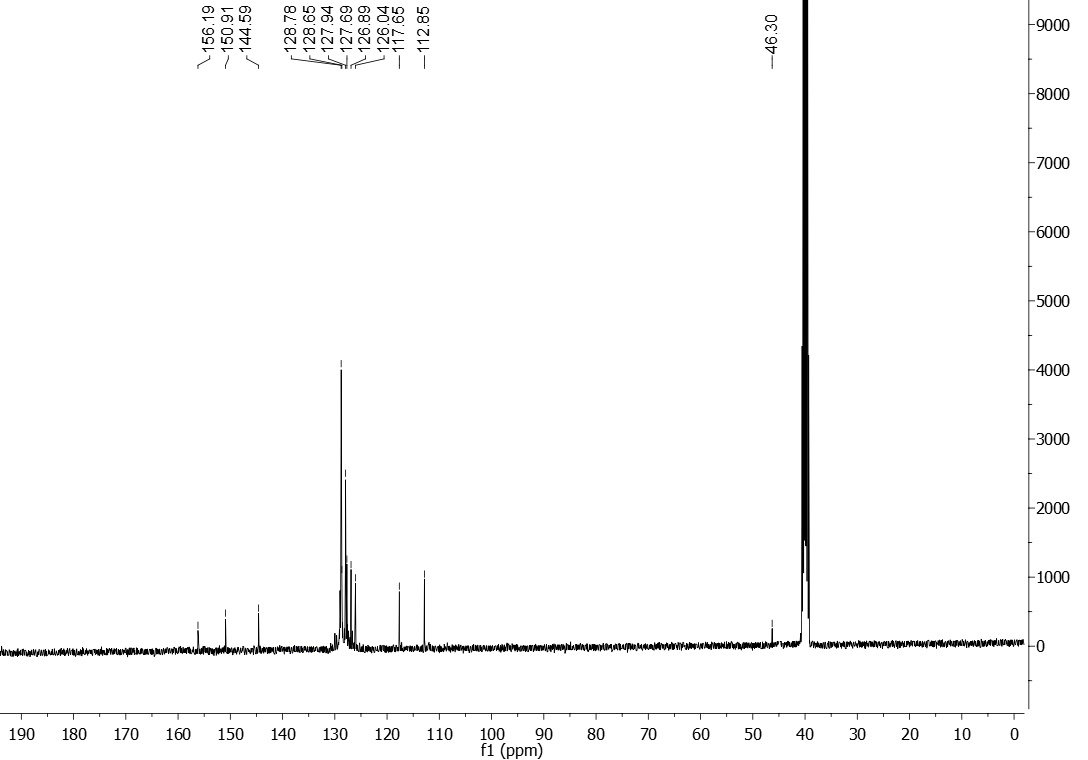
^

**Figure S46. ^13^C NMR (DMSO-*d6*) of (*E*)-1,3-dibenzyl-5-((4-nitrophenyl)diazenyl)pyrimidine-2,4,6(1*H*,3*H*,5*H*)-trione 23**
